# Supplementary material for: Impacts of Inter-annual Wind and Solar Variations on the European Power System
Source: Joule. 2018 Oct 17;2(10):2076–90. doi: 10.1016/j.joule.2018.06.020 (PMC6199136; doi:10.1016/j.joule.2018.06.020)
Supplement: Document S1. Supplemental Experimental Procedures, Figures S1–S5, and Tables S1–S8 [file mmc1.pdf]

JOUL, Volume *2*

Supplemental Information

Impacts of Inter-annual Wind and Solar Variations on the European Power System

Seán Collins, Paul Deane, Brian Ó Gallachóir, Stefan Pfenninger, and Iain Staffell

This document is Creative Commons Attribution 4.0 licensed.

1. AVAILABILITY OF MODELS AND DATA

The PLEXOS model used in this study is available at:

https://energyexemplar.com/datasets/

The Renewables.ninja PV and wind generation dataset is available at:

https://www.renewables.ninja/downloads

2. MODELS AND THEIR ASSUMPTIONS

While all modelling assumptions and data sources underpinning this work have been provided in the manuscript, this section serves to more thoroughly detail the models used to provide further information for the reader regarding the underlying assumptions and implicit limitations of this study.

2.1. PLEXOS Integrated Energy Model

PLEXOS Integrated Energy Model is a power system modelling platform developed by Energy Exemplar that is used for integrated modelling of power, gas and water systems[1]. It is a commercial modelling tool that is free of charge for non-commercial research applications in academic institutions.

In this study, the modelling platform is used to optimise unit commitment and economic dispatch of the power sector by using short term deterministic modelling. The model developed assumes perfect foresight in relation to electrical load, wind and solar production, and forced outages of generators. Stochastic unit commitment is possible within PLEXOS and could be applied to represent imperfect foresight in relation to these parameters. However, this was not applied in this work due to it being outside the scope given the disparity between its typical application in shorter term dispatch planning and our 30 year long period of analysis. This means that while the work is representative of day ahead market modelling, it is idealised and does not represent the imperfect reality of shorter term market function. A perfect day-ahead market is assumed across the EU where there is no market power or anti-competitive bidding behaviour where power stations bid their

true short-run marginal cost, all of which impact the reality of power system operation.

While PLEXOS can be used for optimising investment, this function was not used in this work and only a dispatch simulation of six discreet scenarios were undertaken. Operations planning models, by definition, are not best suited to assessing optimal investment but this work shows how they leveraged to gain insights into the weather induced variability of system operation.

The model minimises the total generation cost of the system while respecting four key constraints: 1) electricity demand and supply must balance; 2) technical characteristics of generators (such as minimum stable levels, ramp rates, minimum up and down times, and maintenance rates); 3) transmission capacity of interconnector lines; 4) forced (random outages based on Monte Carlo simulations) and unforced (scheduled) outages of generators.

PLEXOS models each trading period and ensures chronological consistency across the entire horizon of the optimisation. It models the start-up and shutdown of all generators and tracks them over time. It models the unit commitment and economic dispatch of the power system over the entire time horizon. To ensure consistency across the entire optimisation horizon and avoid problems with inter-temporal constraints at simulation boundary steps, a look ahead period is used which gives the optimiser information about what happens after the period of optimisation. With this look ahead period, the optimiser solves the simulation period and the look ahead period in combination but only keeps the results for the simulation period. In the PLEXOS modelling framework, hydro, wind, solar PV and other renewables are typically treated as free modes of generation with zero marginal cost. This is how they were treated in this study and led them to be dispatched as much as possible subject to resource availability so as to minimise total generation costs.

As mentioned in the manuscript, a core challenge in this work surrounded the acquisition of granular technical characteristic for the ~10,000 power stations across 30 European countries. This led the use of standard generator classes for 15 modes of generation for each country with homogenous characteristics. A summary of the key generator characteristics used in this study is provided below in Table S1. Of course, use of these standard characteristics and standard fuel pricing across Europe will impact results for each scenario. Model results, however, are very sensitive to such differences in generator characteristics and fuel pricing, and unless a comprehensive reliable data source is available, their application would lead to bias in the model results.

Table S1: Standard generator characteristics used in PLEXOS model

Fuel Type	Capacity (MW)	Start Cost (€)	Minimum Stable Generation	Ramp Rate (MW/min)
Biomass-waste fired	300	10,000	30%	30
Derived gasses	150	12,000	40%	30
Geothermal heat	70	3,000	40%	30
Hydro Lakes	150	0	0%	30
Hydro Run of River	200	0	0%	30
Hydrogen plants	300	5,000	40%	30
Natural gas CCGT	450	80,000	40%	30
Natural gas OCGT	100	10,000	20%	30
Nuclear energy	1200	120,000	60%	30
Oil fired	400	75,000	40%	30
Solids fired	300	80,000	30%	30

The model was simulated using the MOSEK solver with rounded relaxation unit commitment, a duality gap of 0.05% and a six hour look ahead. In line with the EU Target Model day-ahead market-scheduling algorithm, known as EUPHEMIA[2], 365 days of the each scenario simulation year were simulated at hourly resolution.

2.2. Renewables.ninja wind and PV generation datasets

The Renewables.ninja PV and wind simulation models[3; 4] were used to generate hourly time series of wind and PV generation aggregated to country levels for 30 historical weather years, from 1985 to 2014.

Renewables.ninja uses the NASA MERRA-2 global meteorological reanalysis[5] to provide consistent weather input data for wind and PV generation. As discussed in Refs.[3; 4] MERRA-2 has many advantages over other global reanalyses, in particular, it provides observations at hourly intervals and has a high spatial resolution of 0.5° latitude and 0.625° longitude[6]. Reanalysis data are known to require bias correction due to systemic errors in the assimilation of data through the underlying weather model, their spatial coarseness and their representation of wind speeds at actual wind farm sites[7]. The Renewables.ninja data are bias-corrected by validation with historic solar PV and wind generation as described in Refs.[3; 4].

Renewables.ninja uses the Global Solar Energy Estimator (GSEE) model[3] for solar PV and the Virtual Wind Farm (VWF) model[8] for wind generation. GSEE was used to simulate PV power output from panels with probabilistic tilt and azimuth angles

drawn from a distribution of known panel angles in Europe, in each MERRA-2 grid cell, the results of which are then aggregated to country level data. The VWF model was used to simulate specific individual wind farms in Europe, both existing and planned, the results of which are aggregated to country level. This is not possible for PV systems due to lacking information about distributed PV installations across Europe.

The resulting bias-corrected datasets show good agreement with reported aggregated generation data (see Refs.[3; 4]). Future work on simulating Europe's decarbonised power system at higher spatial resolutions than the country-aggregated level used here will nevertheless benefit from using newer reanalyses with higher spatial resolution, regional reanalyses, or other more highly resolved datasets such as direct satellite-measured data.

3. EXTENDED RESULTS

3.1. Results Overview: Europe

Table S2: Results overview for Europe

	2015 System	EU Reference 2030	ENTSOE Vision 1 2030	ENTSOE Vision 2 2030	ENTSOE Vision 3 2030	ENTSOE Vision 4 2030
Electricity Price[1] (€/MWh)	44 (32.2%)	82 (32.1%)	68 (31.4%)	60 (32.1%)	60 (33.6%)	64 (33.7%)
Wind-weighted Price (€/MWh)[2]	48 (2.2%)	81 (1.3%)	68 (1.8%)	58 (3.2%)	56 (4.4%)	63 (4.2%)
Solar-weighted Price (€/MWh)	45 (2.8%)	86 (1.7%)	66 (1.9%)	54 (2.6%)	40 (4.5%)	39 (5.7%)
Gas-weighted Price (€/MWh)	69 (2.5%)	92 (2.0%)	96 (1.4%)	105 (2.2%)	95 (1.8%)	99 (2.0%)
Coal-weighed Price (€/MWh)	50 (2.5%)	91 (1.2%)	77 (1.1%)	75 (1.2%)	128 (5.3%)	124 (3.7%)
Nuclear-weighted Price (€/MWh)	40 (2.2%)	75 (1.3%)	61 (1.3%)	54 (2.1%)	61 (3.2%)	66 (3.7%)
Total Generation Cost[3] (€ bn)	47.11 (30.8%)	86.83 (32.1%)	62.09 (32.1%)	44.39 (33.0%)	50.28 (34.2%)	60.47 (34.0%)
Total CO_2 Emissions (Mt)	1001 (31.0%)[4]	917 (31.3%)	713 (32.1%)	551 (33.0%)	233 (35.0%)	288 (34.7%)
Carbon Intensity (gCO_2/kWh)	322.6 (31.0%)	247.8 (31.3%)	209.7 (32.1%)	167.0 (33.0%)	68.5 (35.0%)	80.0 (34.7%)
RE Generation Share	36.7% (31.0%)	47.2% (31.4%)	51.0% (31.3%)	57.0% (31.3%)	68.4% (31.3%)	67.4% (31.3%)
VRE Generation Share	13.4% (32.8%)	24.4% (32.7%)	23.0% (32.9%)	25.2% (33.0%)	35.1% (32.8%)	35.6% (32.7%)
VRE Curtailment	0.1% (326.3%)	0.1% (316.8%)	0.3% (318.5%)	1.6% (314.5%)	4.3% (310.7%)	4% (38.8%)
Interconnector Congestion[5]	26.0% (30.9%)	19.1% (32.6%)	25.1% (32.2%)	28.3% (31.9%)	29.7% (31.0%)	35.0% (30.8%)
Total International Electricity Flow	267 TWh (30.7%)	355 TWh (32.3%)	441 TWh (31.5%)	454 TWh (31.6%)	411 TWh (31.2%)	480 TWh (30.9%)

[1] Wholesale electricity price is defined as the marginal cost of electricity in each region, reflecting the shadow price on the electricity demand-supply constraint. This captures an uplift element to account for start-up costs of thermal plant but excludes taxes, capacity payments or ancillary services. This should be interpreted as an energy-only price in a perfect wholesale market where no market power of strategic behaviours occurs.
[2] Average price received by wind generators (also referred to as 'capture price')
[3] Total Generation Cost = Generation Cost + Start & Shutdown Cost + Emissions Cost
[4] Total electricity emissions from this base year simulation is within 3% of the official verified emissions (1025 Mt) for this year, using our historical 1985-2014 weather data.
[5] Averaged over all transmission lines

3.2. Results Overview: Germany

Table S3: Results overview for Germany

	2015 System	EU Reference 2030	ENTSOE Vision 1 2030	Vision 2 2030	Vision 3 2030	Vision 4 2030
Electricity Price (€/MWh)	46 (36.5%)	105 (31.9%)	78 (32.5%)	73 (32.3%)	59 (35.1%)	68 (34.6%)
Wind-weighted Price (€/MWh)	41 (6.0%)	95 (2.3%)	67 (3.7%)	64 (3.5%)	45 (7.0%)	53 (6.0%)
Solar-weighted Price (€/MWh)	39 (7.8%)	114 (2.8%)	70 (3.3%)	58 (3.9%)	39 (5.7%)	45 (6.9%)
Gas-weighted Price (€/MWh)	99 (48.8%)	115 (3.2%)	105 (5.6%)	101 (4.3%)	89 (2.5%)	93 (2.2%)
Coal-weighed Price (€/MWh)	47 (5.7%)	104 (1.9%)	83 (1.7%)	79 (1.5%)	107 (23.3%)	118 (14.4%)
Nuclear-weighted Price (€/MWh)	45 (6.2%)	0 (0%)	0 (0%)	0 (0%)	0 (0%)	0 (0%)
Total Generation Cost (€ bn)	9.16 (31.7%)	19.70 (32.2%)	12.61 (33.0%)	10.77 (33.2%)	7.66 (37.8%)	10.01 (36.8%)
Total CO_2 Emissions (Mt)	270 (31.9%)	288 (31.0%)	221 (32.7%)	190 (33.3%)	38 (38.6%)	50 (37.8%)
Carbon Intensity (gCO_2/kWh)	509.1 (31.9%)	455.5 (31.5%)	372.0 (33.3%)	369.3 (33.5%)	70.7 (39.6%)	91.1 (38.7%)
RE Generation Share	30.2% (33.3%)	45.4% (32.6%)	57.6% (32.6%)	58.5% (32.7%)	82.2% (32.0%)	77.6% (32.4%)
VRE Generation Share	22.7% (34.4%)	36.8% (33.4%)	41.2% (33.9%)	37.2% (34.3%)	58.0% (33.3%)	56.5% (33.6%)
VRE Curtailment	0.0% (30%)	0.0% (30%)	0.4% (327.4%)	0.3% (328.7%)	7.9% (312.4%)	7.9% (38.2%)
Interconnector Congestion	25.1% (31.8%)	16.4% (34.2%)	24.4% (34.0%)	22.3% (33.6%)	31.7% (32.1%)	32.6% (31.9%)
Total International Electricity Flow	53 TWh (31.6%)	79 TWh (33.6%)	105 TWh (33.0%)	100 TWh (32.7%)	109 TWh (31.9%)	121 TWh (31.6%)

3.3. Results Overview: Spain

Table S4 - Results overview for Spain

	2015 System	EU Reference 2030	ENTSOE Vision 1 2030	Vision 2 2030	Vision 3 2030	Vision 4 2030
Electricity Price (€/MWh)	52 (31.9%)	81 (1.4%)	76 (31.5%)	68 (32.3%)	71 (33.5%)	68 (34.9%)
Wind-weighted Price (€/MWh)	47 (2.7%)	76 (1.6%)	69 (1.6%)	63 (2.9%)	64 (3.8%)	63 (5.1%)
Solar-weighted Price (€/MWh)	49 (1.6%)	72 (1.4%)	64 (1.8%)	43 (3.3%)	48 (4.4%)	30 (6.4%)
Gas-weighted Price (€/MWh)	57 (1.9%)	88 (2.6%)	95 (2.7%)	100 (3.4%)	85 (2.7%)	91 (3.9%)
Coal-weighed Price (€/MWh)	52 (1.9%)	83 (1.1%)	79 (1.3%)	79 (1.8%)	92 (27.2%)	113 (24.5%)
Nuclear-weighted Price (€/MWh)	51 (1.8%)	81 (1.3%)	75 (1.3%)	69 (2.4%)	72 (3.5%)	73 (4.8%)
Total Generation Cost (€ bn)	4.03 (32.3%)	6.42 (33.5%)	5.92 (32.9%)	4.23 (33.5%)	8.88 (32.5%)	8.85 (32.5%)
Total CO_2 Emissions (Mt)	76 (32.2%)	50 (32.5%)	66 (32.1%)	48 (33.1%)	42 (33.0%)	39 (33.2%)
Carbon Intensity (gCO_2/kWh)	315.4 (32.3%)	182.1 (32.6%)	221.8 (32.3%)	168.5 (33.1%)	121.3 (33.0%)	104.1 (33.2%)
RE Generation Share	42.0% (32.5%)	52.7% (32.1%)	52.0% (31.8%)	61.4% (31.3%)	55.9% (31.6%)	61.7% (31.4%)
VRE Generation Share	25.1% (34.2%)	36.8% (33.2%)	34.7% (32.9%)	38.5% (32.2%)	35.9% (32.7%)	43.1% (32.0%)
VRE Curtailment	0.2% (352.5%)	0.1% (345.4%)	0.2% (336.8%)	1.2% (315.5%)	0.8% (320.9%)	0.8% (356.2%)
Interconnector Congestion	20.2% (37.2%)	9.6% (312.4%)	11.2% (310.6%)	18.1% (37.0%)	11.3% (311.6%)	6.6% (313.5%)
Total International Electricity Flow	10 TWh (34.1%)	28 TWh (35.5%)	34 TWh (34.0%)	37 TWh (33.6%)	28 TWh (35.8%)	17TWh (36.8%)

3.4. Results Overview: France

Table S5: Results overview for France

	2015 System	EU Reference 2030	ENTSOE Vision 1 2030	Vision 2 2030	Vision 3 2030	Vision 4 2030
Electricity Price (€/MWh)	38 (32.3%)	74 (31.7%)	59 (31.4%)	53 (32.5%)	60 (34.2%)	64 (35.0%)
Wind-weighted Price (€/MWh)	36 (3.2%)	70 (1.5%)	56 (1.8%)	49 (3.5%)	51 (5.6%)	55 (6.6%)
Solar-weighted Price (€/MWh)	34 (1.7%)	71 (1.1%)	54 (1.5%)	44 (3.2%)	39 (5.9%)	38 (6.5%)
Gas-weighted Price (€/MWh)	77 (14.7%)	98 (12.4%)	97 (12.5%)	93 (6.6%)	86 (3.4%)	89 (3.6%)
Coal-weighed Price (€/MWh)	44 (2.9%)	75 (1.1%)	64 (1.3%)	64 (2.1%)	72 (19.6%)	89 (23.5%)
Nuclear-weighted Price (€/MWh)	37 (2.0%)	74 (1.5%)	60 (1.3%)	53 (2.5%)	62 (3.9%)	66 (4.7%)
Total Generation Cost (€ bn)	2.97 (30.9%)	4.18 (31.6%)	3.27 (31.4%)	2.88 (31.4%)	3.59 (35.3%)	4.18 (35.8%)
Total CO_2 Emissions (Mt)	16 (33.8%)	29 (32.1%)	16 (33.7%)	10 (35.8%)	11 (39.2%)	13 (39.1%)
Carbon Intensity (gCO_2/kWh)	29.9 (33.8%)	46.6 (32.4%)	29.7 (33.9%)	18.5 (35.8%)	21.5 (39.2%)	25.8 (39.3%)
RE Generation Share	19.9% (30.9%)	32.8% (31.2%)	27.4% (31.2%)	25.1% (30.9%)	45.2% (31.3%)	46.4% (31.6%)
VRE Generation Share	5.4% (33.5%)	18.1% (32.5%)	13.1% (32.8%)	8.3% (32.9%)	26.2% (32.5%)	28.2% (32.8%)
VRE Curtailment	0.0% (30%)	0.0% (30%)	0.1% (356.6%)	0.5% (342.0%)	1.2% (323.5%)	1.2% (327.4%)
Interconnector Congestion	47.1% (31.8%)	46.3% (32.7%)	47.4% (32.5%)	41.3% (31.9%)	23.1% (34.8%)	22.7% (34.8%)
Total International Electricity Flow	47 TWh (31.1%)	81 TWh (32.7%)	92 TWh (32.4%)	75 TWh (31.5%)	57 TWh (35%)	46 TWh (35.2%)

3.5. Results Overview: Italy

Table S6: Results overview for Italy

	2015 System	EU Reference 2030	ENTSOE Vision 1 2030	Vision 2 2030	Vision 3 2030	Vision 4 2030
Electricity Price (€/MWh)	58 (30.9%)	85 (31.1%)	90 (31.0%)	83 (31.6%)	72 (32.2%)	76 (33.1%)
Wind-weighted Price (€/MWh)	55 (1.0%)	85 (1.4%)	85 (1.3%)	77 (2.1%)	64 (3.8%)	68 (3.1%)
Solar-weighted Price (€/MWh)	56 (1.2%)	82 (0.8%)	76 (1.4%)	65 (2.0%)	39 (3.9%)	44 (5.3%)
Gas-weighted Price (€/MWh)	60 (1.1%)	90 (2.0%)	108 (1.5%)	112 (1.7%)	94 (2.0%)	93 (2.2%)
Coal-weighed Price (€/MWh)	56 (0.9%)	86 (1.1%)	88 (1.0%)	84 (1.3%)	100 (22.7%)	110 (18.1%)
Nuclear-weighted Price (€/MWh)	0 (0%)	0 (0%)	0 (0%)	0 (0%)	0 (0%)	0 (0%)
Total Generation Cost (€ bn)	6.93 (30.8%)	11.7 (31.4%)	9.93 (31.0%)	7.18 (31.6%)	7.78 (32.3%)	10.09 (32.1%)
Total CO_2 Emissions (Mt)	97 (30.5%)	80 (31.0%)	94 (30.8%)	77 (31.3%)	38 (32.9%)	49 (32.5%)
Carbon Intensity (gCO_2/kWh)	362.3 (30.5%)	272.2 (30.9%)	305.8 (30.6%)	263.6 (30.9%)	129.0 (32.3%)	147.6 (32.0%)
RE Generation Share	41.2% (31.0%)	46.6% (31.3%)	49.5% (30.9%)	59.6% (30.8%)	55.9% (31.6%)	69.2% (31.0%)
VRE Generation Share	15.3% (32.7%)	21.5% (32.9%)	34.7% (32.9%)	21.3% (32.1%)	35.9% (32.7%)	30.3% (32.1%)
VRE Curtailment	0% (30%)	0% (30%)	0% (30%)	0.5% (320.8%)	4.2% (38.6%)	4.2% (37.8%)
Interconnector Congestion	48.9% (32.1%)	60.1% (32.4%)	48.1% (32.3%)	36.6% (34.3%)	37.8% (33.9%)	42.2% (4.2%)
Total International Electricity Flow	48 TWh (31.3%)	61 TWh (32.9%)	49 TWh (33.1%)	45 TWh (34.1%)	11 TWh (38.0%)	31TWh (36.3%)

3.6. Results Overview: Great Britain

Table S7: Results overview for Great Britain

	2015 System	EU Reference 2030	ENTSOE Vision 1 2030	Vision 2 2030	Vision 3 2030	Vision 4 2030
Electricity Price (€/MWh)	59 (31.6%)	79 (31.3%)	81 (31.2%)	55 (35.4%)	60 (34.5%)	61 (35.2%)
Wind-weighted Price (€/MWh)	56 (31.9%)	76 (31.3%)	77 (31.3%)	43 (36.7%)	47 (35.7%)	47 (36.3%)
Solar-weighted Price (€/MWh)	54 (32.9%)	78 (31.4%)	77 (31.9%)	54 (35.0%)	43 (35.8%)	47 (35.7%)
Gas-weighted Price (€/MWh)	67 (31.5%)	84 (31.8%)	86 (1.4%)	83 (4.1%)	87 (2.5%)	89 (2.7%)
Coal-weighed Price (€/MWh)	59 (31.6%)	80 (31.0%)	83 (31.3%)	78 (33.5%)	47 (35.7%)	0 (30%)
Nuclear-weighted Price (€/MWh)	58 (31.4%)	79 (31.2%)	80 (31.2%)	60 (33.7%)	66 (33.0%)	68 (33.5%)
Total Generation Cost (€ bn)	6.56 (31.5%)	8.82 (35.5%)	10.46 (32.8%)	3.56 (310.5%)	6.19 (37.9%)	6.60 (38.7%)
Total CO_2 Emissions (Mt)	146 (31.3%)	40 (36.0%)	70 (32.9%)	25 (310.6%)	28 (39.2%)	29 (39.8%)
Carbon Intensity (gCO_2/kWh)	523.2 (31.3%)	109.7 (36.0%)	249.9 (32.8%)	80.4 (311.8%)	76.6 (39.5%)	77.5 (310.2%)
RE Generation Share	22.2% (34.2%)	49.9% (33.4%)	32.9% (35.0%)	76.3% (32.9%)	65.6% (33.6%)	67.4% (33.6%)
VRE Generation Share	17.7% (35.6%)	31.9% (35.4%)	27.3% (36.0%)	64.3% (33.7%)	52.5% (34.7%)	56.6% (34.4%)
VRE Curtailment	0% (30%)	0% (30%)	0% (30%)	4.3% (315.7%)	2.7% (316.9%)	2.7% (318.0%)
Interconnector Congestion	35.9% (32.0%)	29.8% (33.2%)	39.9% (32.3%)	51.0% (32.3%)	36.3% (32.0%)	44.0% (1.6%)
Total International Electricity Flow	18 TWh (31.6%)	42 TWh (33.1%)	58 TWh (31.7%)	60 TWh (31.7%)	45 TWh (31.7%)	52TWh (31.5%)

4. WIND AND SOLAR OUTPUT VARIABILITY

This section shows the variability of annual capacity factors for wind and solar profiles by country for all scenarios considered. For all diagrams, the text on each country describes the mean capacity factor followed by the percentage point standard deviation over the course of all 30 weather years. The colour scale indicates the mean capacity factor for either wind or solar PV in each country.

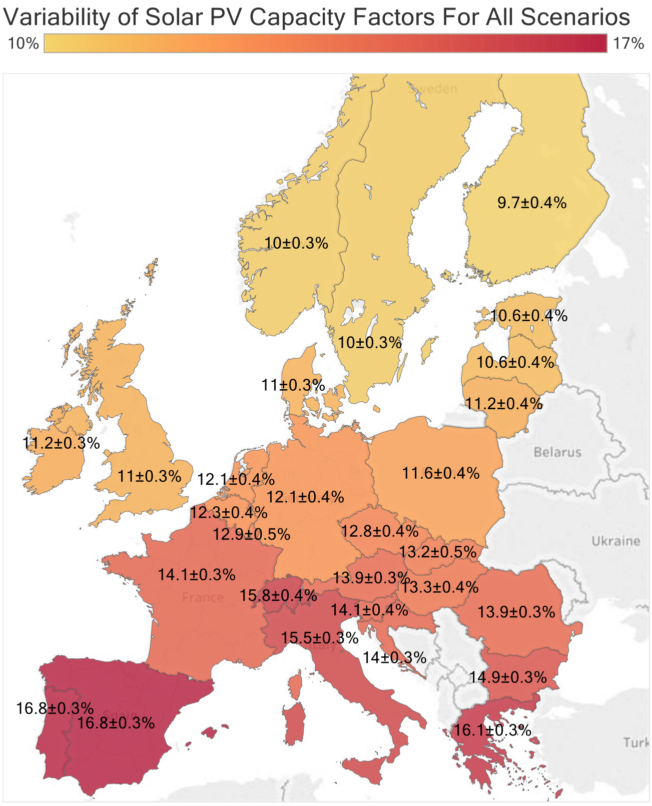

Figure S1: Solar capacity factor variability for all scenarios considered

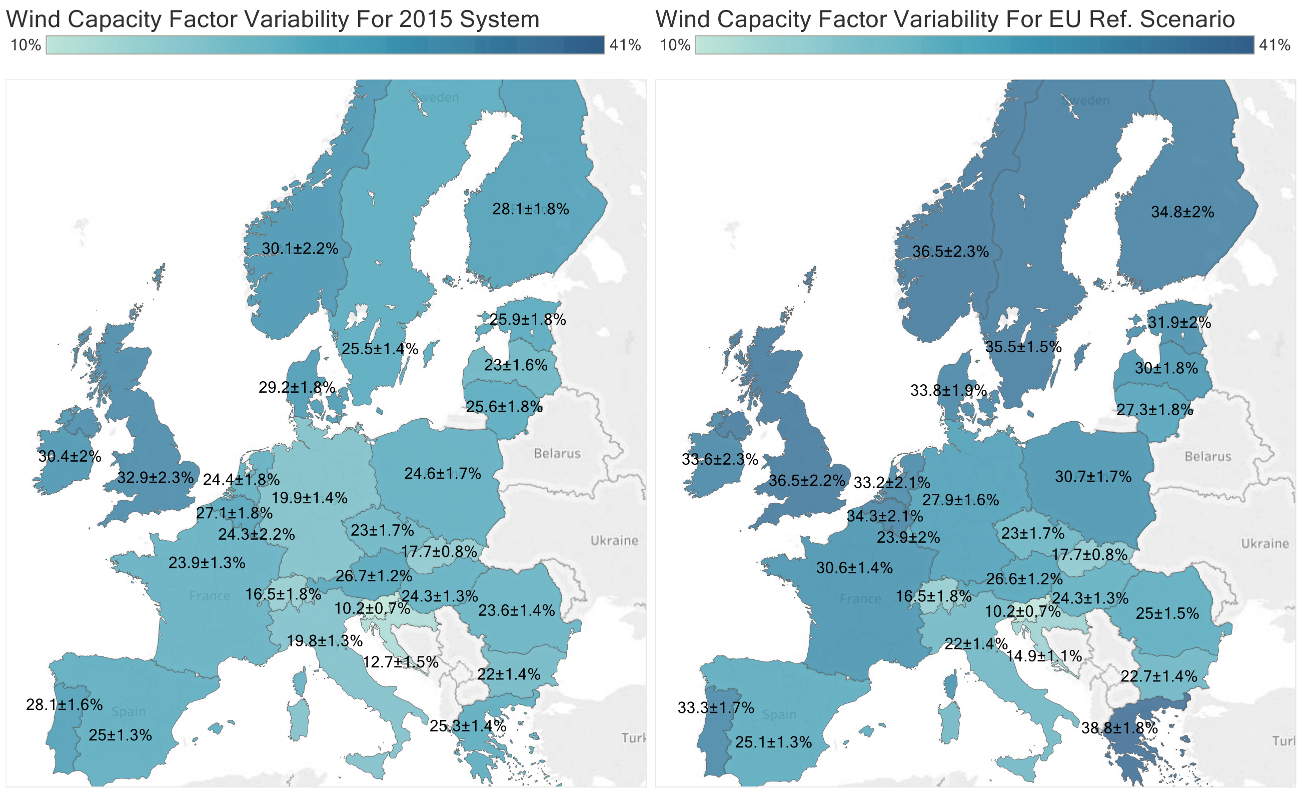

Figure S2: Wind capacity factor variability for 2015 System and EU Reference Scenario

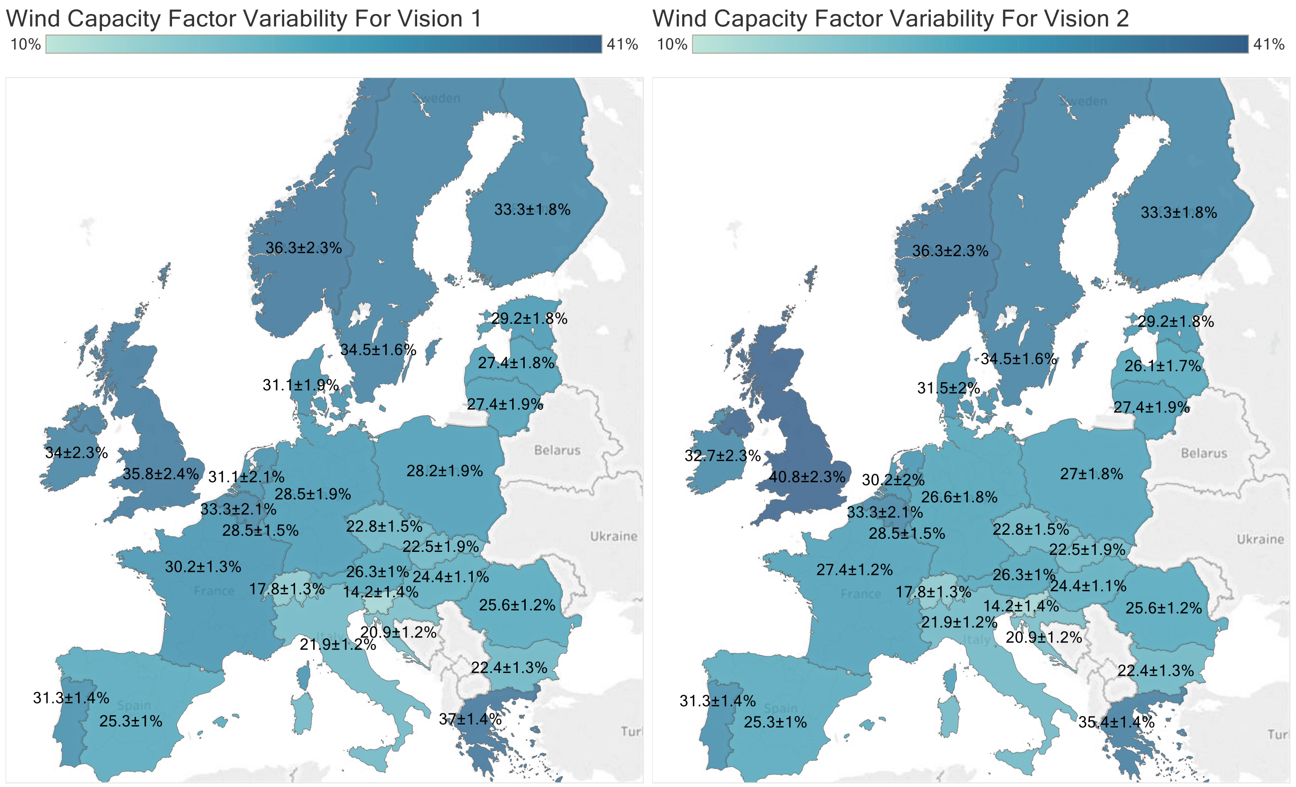

Figure S3: Wind capacity factor variability for Vision 1 and Vision 2

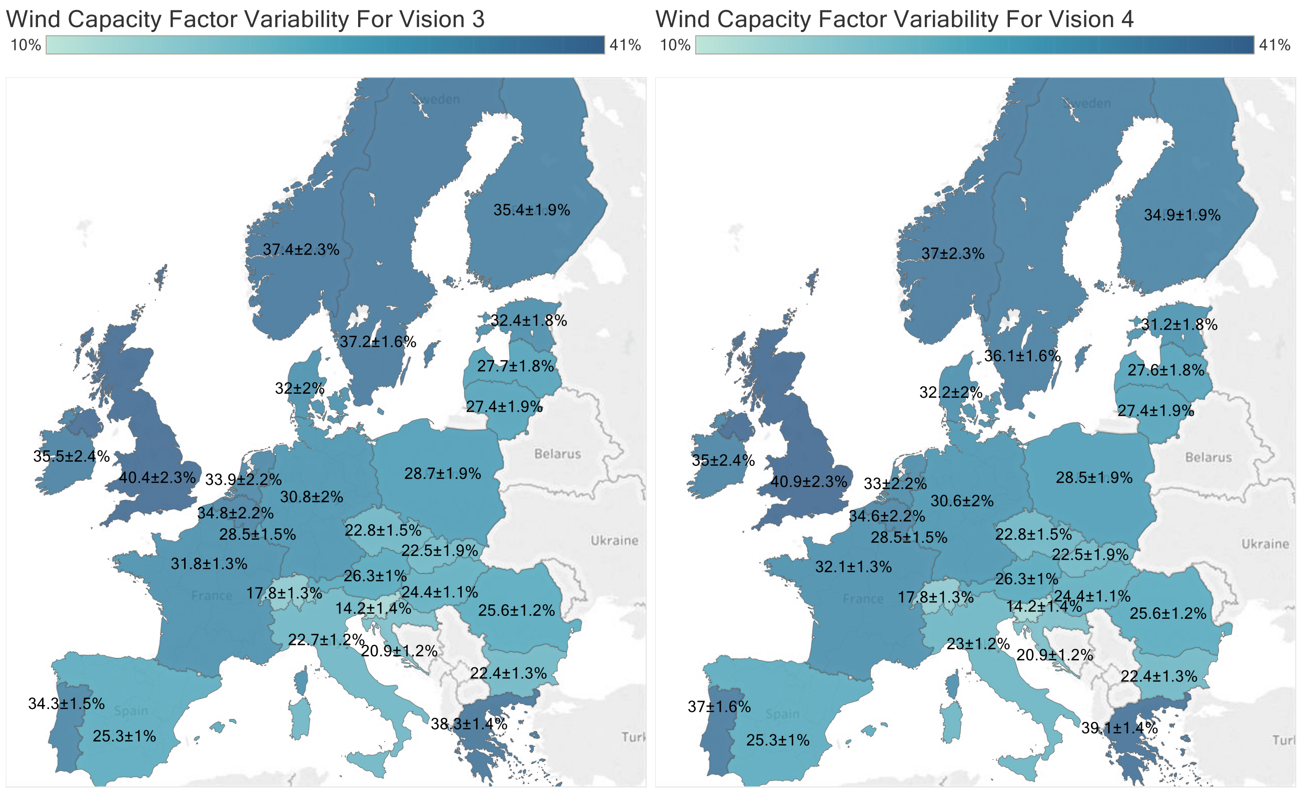

Figure S4: Wind capacity factor variability for Vision 3 and Vision 4

5. MOST REPRESENTATIVE SINGLE YEARS

The below table details the root mean squared error across eight metrics for all weather years considered. This error is determined as the RMS difference between each year and the long-run mean for each metric normalised by the long-run mean.

Table S8: Root mean squared error across various metrics for all weather years

Weather Year	VRE Penetratio n	CO_2 Emissions	Total Generatio n Costs	Market Prices	RE Penetratio n	IC Congestion	Total IC Flow	VRE Curtailmen t	Average RMS Error
1985	3.4%	3.7%	3.4%	1.2%	0.6%	2.2%	1.6%	3.3%	2.43%
1986	2.8%	2.6%	2.9%	1.2%	1.5%	0.7%	0.5%	12.3%	3.05%
1987	4.8%	4.8%	4.4%	1.9%	1.6%	1.7%	1.8%	12.3%	4.16%
1988	2.5%	2.7%	2.6%	1.2%	1.9%	1.2%	0.9%	3.9%	2.11%
1989	0.7%	0.6%	0.5%	0.9%	0.8%	1.0%	0.5%	0.9%	0.72%
1990	4.8%	4.7%	4.4%	2.8%	1.0%	2.4%	2.3%	24.2%	5.82%
1991	1.2%	1.3%	1.2%	1.0%	1.6%	0.3%	0.4%	9.2%	2.04%
1992	2.0%	1.8%	1.6%	1.1%	0.7%	0.7%	0.3%	16.4%	3.08%
1993	2.1%	2.2%	2.1%	1.5%	0.9%	1.2%	1.3%	7.3%	2.32%
1994	3.6%	3.4%	3.2%	2.8%	1.1%	1.5%	1.3%	19.2%	4.50%
1995	3.4%	3.3%	3.3%	2.5%	1.6%	1.9%	1.4%	1.3%	2.32%
1996	1.1%	1.4%	0.7%	1.0%	1.2%	0.7%	0.6%	14.4%	2.65%
1997	2.5%	2.8%	2.8%	1.3%	0.8%	0.9%	0.5%	8.5%	2.52%
1998	4.8%	4.9%	4.5%	2.9%	1.5%	1.8%	1.8%	12.3%	4.32%
1999	2.1%	1.8%	2.0%	1.4%	1.7%	1.1%	0.6%	3.7%	1.80%
2000	3.0%	2.9%	2.7%	1.4%	1.1%	2.2%	1.7%	2.0%	2.13%
2001	1.2%	1.3%	0.9%	0.5%	1.1%	1.1%	1.0%	4.9%	1.50%
2002	0.5%	0.7%	0.6%	0.6%	0.4%	0.7%	0.7%	4.3%	1.07%
2003	3.7%	3.9%	3.5%	2.1%	0.8%	2.4%	2.4%	9.6%	3.55%
2004	0.9%	0.9%	1.1%	0.7%	1.3%	0.6%	0.3%	7.4%	1.64%
2005	0.6%	0.8%	0.6%	0.5%	0.3%	0.5%	1.0%	6.3%	1.34%
2006	2.9%	2.7%	2.6%	2.0%	0.6%	1.6%	1.3%	8.4%	2.76%
2007	1.6%	1.7%	1.4%	1.4%	1.1%	2.0%	1.9%	2.8%	1.73%
2008	1.6%	2.1%	2.1%	1.7%	0.8%	0.7%	0.8%	4.3%	1.77%
2009	2.5%	2.6%	2.2%	1.3%	0.9%	1.8%	1.5%	9.3%	2.75%
2010	5.9%	5.6%	5.3%	3.1%	1.7%	2.2%	1.8%	26.1%	6.45%
2011	0.4%	0.3%	0.3%	0.6%	1.8%	0.5%	0.4%	7.3%	1.44%
2012	0.9%	0.6%	0.7%	0.6%	0.3%	0.4%	0.3%	3.7%	0.92%
2013	1.1%	1.3%	1.0%	0.5%	0.5%	1.1%	0.6%	3.9%	1.24%
2014	2.0%	1.5%	3.2%	8.1%	0.6%	0.5%	0.7%	12.0%	3.58%

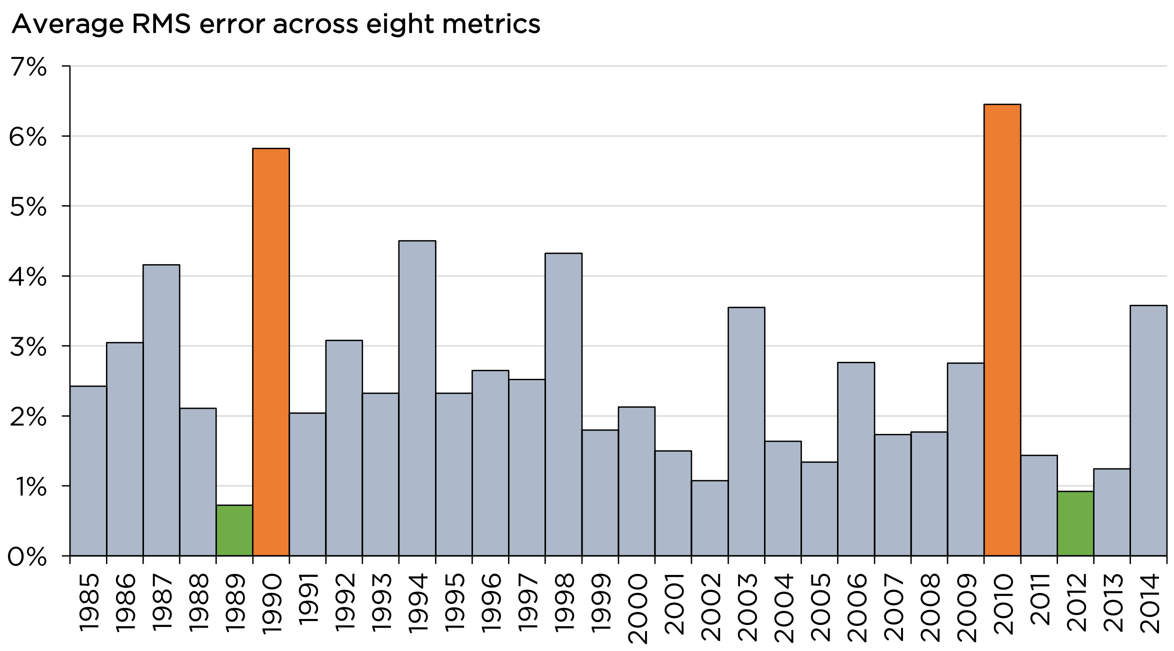

Figure S5: Average RMS Error across eight metrics

6. REFERENCES

1 Energy-Exemplar (2016). PLEXOS Integrated Energy Model. (http://energyexemplar.com/software/plexos-desktop-edition/).

2 Price Coupling of Regions (2016). EUPHEMIA: Description and Functioning, in: PCR (Ed.), EUPHEMIA Stakeholder Forum. EPEX Spot, Brussels. (http://static.epexspot.com/document/37602/Euphemia%20Public%20Presentation).

3 Pfenninger, S., and Staffell, I. (2016). Long-term patterns of European PV output using 30 years of validated hourly reanalysis and satellite data. Energy *114*, 1251-1265.

4 Staffell, I., and Pfenninger, S. (2016). Using bias-corrected reanalysis to simulate current and future wind power output. Energy *114*, 1224-1239.

5 Gelaro, R., McCarty, W., Suárez, M.J., Todling, R., Molod, A., Takacs, L., Randles, C.A., Darmenov, A., Bosilovich, M.G., Reichle, R., *et al.* (2017). The Modern-Era Retrospective Analysis for Research and Applications, Version 2 (MERRA-2). Journal of Climate *30*, 5419-5454.

6 Liléo, S., and Petrik, O. (2000). Investigation on the use of NCEP/NCAR, MERRA and NCEP/CFSR reanalysis data in wind resource analysis. sigma *1*.

7 Stickler, A., and Brönnimann, S. (2011). Significant bias of the NCEP/NCAR and twentieth-century reanalyses relative to pilot balloon observations over the West African Monsoon region (1940–1957). Quarterly Journal of the Royal Meteorological Society *137*, 1400-1416.

8 Staffell, I., and Green, R. (2014). How does wind farm performance decline with age? Renewable energy *66*, 775-786.
