# Supplementary material for: Impacts of Inter-annual Wind and Solar Variations on the European Power System
Source: Joule. 2018 Oct 17;2(10):2076–90. doi: 10.1016/j.joule.2018.06.020 (PMC6199136; doi:10.1016/j.joule.2018.06.020)
Supplement: Document S2. Article plus Supplemental Information [file mmc2.pdf]

Joule

CellPress

Article

Impacts of Inter-annual Wind and Solar Variations on the European Power System

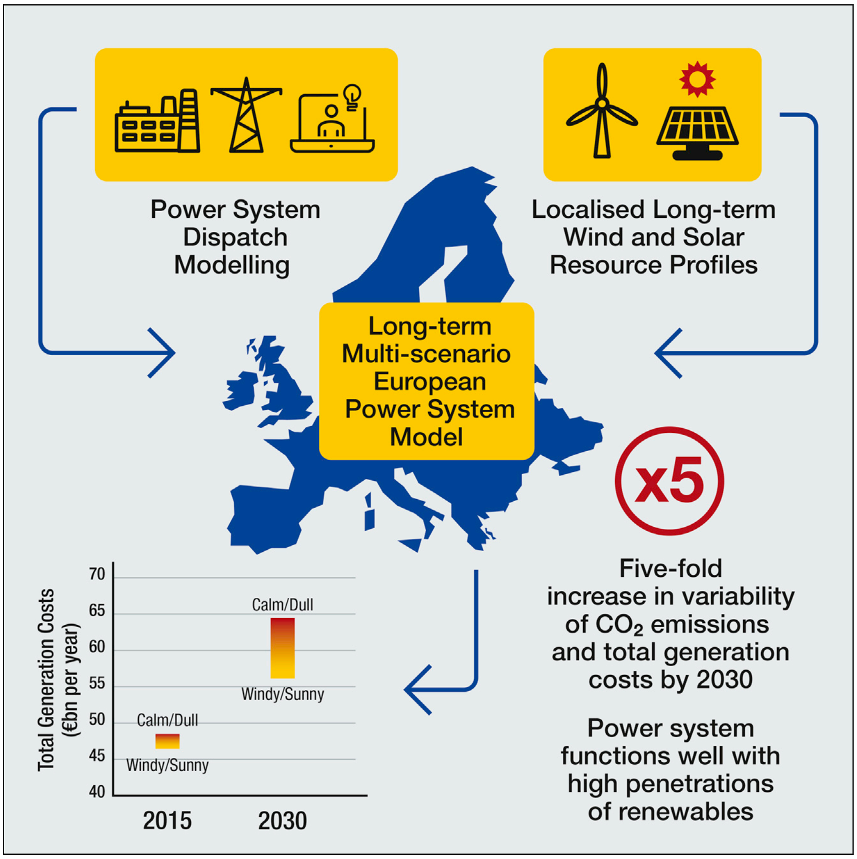

This research sheds light on the impact of long-term weather variability on the operation of the European power system and how this scales with uptake of wind and solar power out to 2030. We find that ambitious decarbonization leads to much greater influence of long-term weather patterns, with a 5-fold increase in operational variability by 2030. Several relevant metrics can be reasonably approximated by linear functions of variable renewable penetration, providing a shortcut for estimating the impacts of intermittency.

Seán Collins, Paul Deane, Brian Ó Gallachóir, Stefan Pfenninger, Iain Staffell

sean.collins@umail.ucc.ie

HIGHLIGHTS

The impact of weather patterns on power system increases with decarbonization

Europe's CO_2 output and generation cost variability could increase 5-fold by 2030

Several metrics can be reasonably approximated from the level VRE penetration

The most representative single years for renewable generation are 1989 and 2012

Collins et al., Joule *2*, 2076–2090
October 17, 2018 © 2018 The Authors.
Published by Elsevier Inc.
https://doi.org/10.1016/j.joule.2018.06.020

Check for updates

Joule

CellPress

Article

Impacts of Inter-annual Wind and Solar Variations on the European Power System

Seán Collins,[1,2,5,*] Paul Deane,[1,2] Brian Ó Gallachóir,[1,2] Stefan Pfenninger,[3] and Iain Staffell[4]

SUMMARY

Weather-dependent renewable energy resources are playing a key role in decarbonizing electricity. There is a growing body of analysis on the impacts of wind and solar variability on power system operation. Existing studies tend to use a single or typical year of generation data, which overlooks the substantial year-to-year fluctuation in weather, or to only consider variation in the meteorological inputs, which overlooks the complex response of an interconnected power system. Here, we address these gaps by combining detailed continent-wide modeling of Europe's future power system with 30 years of historical weather data. The most representative single years are 1989 and 2012, but using multiple years reveals a 5-fold increase in Europe's inter-annual variability of CO_2 emissions and total generation costs from 2015 to 2030. We also find that several metrics generalize to linear functions of variable renewable penetration: CO_2 emissions, curtailment of renewables, wholesale prices, and total system costs.

Context & Scale

Wind and solar power have been driving the decarbonization of Europe's electricity over the last decade. Increasing our reliance on weather-dependent resources makes it imperative that planning of electricity systems becomes cognizant of their long-term variability. Studies often neglect the long-term variability of these resources by using only data from a single or a few years or fail to account for the impacts of short-term international electricity flows and limitations on generator flexibility, which are critical to the integration of these variable generation sources.

This study uses a continental electricity system model and 30 years of hourly wind and solar data to determine the impact of long-term weather patterns on European electricity system operation and how this varies with decarbonization ambition. The results show that the variability of CO_2 emissions and total generation costs for this interconnected electricity system could increase 5-fold by 2030 compared with 2015.

INTRODUCTION

Variable renewable energy (VRE) technologies, namely wind and solar photovoltaics (PVs), have grown over 4-fold in capacity in Europe over the last decade from 62 GW in 2007 to 260 GW in 2016[1] and are reducing power sector emissions worldwide. However, their effects on system operation include reduced market pricing, increased interconnector flows, greater need for balancing, as well as reserve and curtailment of renewable power.[2–7] Long-term energy system models, used to project technology pathways for policy development, struggle to capture climatic variability and thus poorly represent challenges associated with decarbonization of the electricity sector.[8,9] Many studies use a single or small number of years of meteorological data, which neglects the impact of long-term temporal variability of weather on the power sector.[10–14] Many studies also focus on a single country or small regions,[15–19] which neglects the corresponding impact of spatial variability. Crucially, this neglects the large-scale temporal and spatial variations and correlations seen in weather systems.[20–23] Insufficient temporal and spatial resolution within these models means that the operational challenges of such variability are not sufficiently captured, regardless of the quality of the input data.[8,24,25]

Various methods have been developed to address limitations of long-term energy system models in capturing wind and solar variability.[26,27] Studies are beginning to make use of longer-term and more spatially explicit datasets. For example, Bloomfield et al.[28] and Pfenninger[27] both consider 25 years of weather data within the UK to explore variability in optimal generation investments, but considering a single country in isolation neglects the potential for balancing renewable intermittency through international trade. Shaner et al.,[29] Olauson et al.,[30] Burtin and Silva,[31]

 © 2018 The Authors. Published by Elsevier Inc.
This is an open access article under the CC BY license (http://creativecommons.org/licenses/by/4.0/).

and Grams et al.[32] combine long-term datasets with wider geographic scope (The United States, Scandinavia, and Europe), but in their analyses of long-term variability they only explore the statistical properties of demand net of renewable output, ignoring the constrained responses of real power systems. Existing work fails to explore the full extent of renewable variability impacts across a continent-scale electricity system. Without modeling the limited interconnection between countries, the flexibility of conventional generators, and the cost of backup capacity, implications of increasing variable renewable generation, such as cost and carbon emissions, are therefore not yet fully understood. The recent controversy surrounding Jacobson et al.'s[33] and Clack et al.'s[34] divergent views on the decarbonized US power system underscores the importance of model assumptions on results. It illustrates how closed and opaque modeling harms the credibility of work in this field[35] and prevents users and readers from fully understanding the limitations of model outputs.[36] Here, we address all these gaps by performing a multi-scenario analysis of the European power system with an industry standard power system dispatch model using 30 years of wind and solar profiles developed using open-access weather data. Our complete model is openly available (see https://www.renewables.ninja/downloads and https://energyexemplar.com/datasets/ for the PLEXOS model).

Ideally, such a study would also incorporate long-term variability in hydro generation (due to precipitation) and electricity demand (due to temperature). However, these are nascent areas of research so they cannot yet be modeled with sufficient confidence at the continental scale to generate meaningful results (unlike wind and solar).[37,38] The impact of longer-term climate change on variability of renewable resources also merits consideration but current thinking suggests this will be insignificant over Europe within the time horizon of this study.[39–44]

Modeling

We use a pan-European electricity dispatch model developed in PLEXOS,[45] which captures power station characteristics and constrained transmission of power between countries.[46] We model the least-cost dispatch of electricity under several levels of decarbonization ambition across 29 countries at hourly resolution while respecting the technical constraints of generators and levels of international transmission capacity. We run the model for a 2015 baseline system and five official scenarios that define electricity demand, renewable energy penetration, and the installed fleet of power stations in 2030. Together, these show how system operation changes with decarbonization ambition. The future scenarios are based on the European Commission's EU Reference Scenario[47] and the European Network of Transmission System Operators for Electricity's (ENTSO-E's) four "visions" used to inform the 10-year network development plan.[48] These possible futures encompass a broad range of ambition toward achieving the EU 2050 Roadmap sustainability goals, which translates to various penetrations of different technologies (particularly VRE generation) across the scenarios considered. In terms of electricity demand, this translates to the wide range of demand response, electric vehicle penetration, and electrification of heating, all of which are endogenous in the demand profiles used. An overview of all these scenarios is shown in Table 1 and are further detailed in[47] and.[48]

These six power system scenarios were modeled with 30 years of synthesized hourly output (1985–2014) from each country's wind and solar fleet, derived from the Renewables.ninja models.[49,50] These output profiles differ between scenarios due to the assumed wind capacity and share of onshore and offshore. The productivity of German wind farms, for example, ranged from 19.9% in 2015 to between 26.6%

[1]MaREI Centre, Environmental Research Institute, University College Cork, Lee Road, Cork, Ireland

[2]School of Engineering, University College Cork, Cork, Ireland

[3]Climate Policy Group, Institute for Environmental Decisions, Zurich ETH 8092, Switzerland

[4]Centre for Environmental Policy, Imperial College London, London SW7 1NA, UK

[5]Lead Contact

*Correspondence: sean.collins@umail.ucc.ie

https://doi.org/10.1016/j.joule.2018.06.020

Table 1. Comparison of Scenarios Considered in This Work

	2015 System	EU Reference 2030	Vision 1 2030	Vision 2 2030	Vision 3 2030	Vision 4 2030
Electricity demand (TWh)	3,103	3,752	3,434	3,251	3,376	3,616
Variable renewable capacity (GW)	241	447	388	390	572	614
Fuel prices (€/GJ)						
Natural gas	6.6	9.7	9.5	9.5	7.2	7.2
Oil	8.2	16	17.3	17.3	13.3	13.3
Coal	2	3.5	3.0	3.0	2.8	2.2
CO_2 price (€/tonne)	7.5	32	17	17	71	76
Merit order	coal before gas	coal before gas	coal before gas	coal before gas	gas before coal	gas before coal

Variable renewable generation sources discussed in the context of this work consist of wind and solar PV generation only.

and 30.8% in 2030. Further information regarding the methodology, models, and data used (including maps displaying the mean and inter-annual variability of these wind and solar profiles) can be found in the Experimental Procedures section and in the Supplemental Information.

RESULTS

Power System Evolution under Different Degrees of Ambition

The scenarios we use assume that energy sector decarbonization is achieved primarily by increasing the share of variable renewable generation, rather than other options such as nuclear or carbon capture and storage. Table 2 provides an overview of how the operation of the power sector changes with different degrees of decarbonization ambition under these scenarios (i.e., different amounts of VRE deployment) and quantifies how year-to-year variations in weather patterns affect the power sector's operation. Table 2 displays results for three scenarios. The mean of each metric is listed followed by its coefficient of variation across all weather years in brackets. Wholesale electricity price is defined as the marginal cost of electricity in each region, reflecting the shadow price on the electricity demand-supply constraint. This captures an uplift element to account for startup costs of thermal plant but excludes taxes, capacity payments, or ancillary services. Scarcity pricing (a price cap in the event of unserved energy) was used in the model in the determination of regional wholesale energy prices. This should be interpreted as an energy-only price in a perfect wholesale market where no market power or strategic behaviors occurs. The absence of market power is a key aim of the European internal electricity market and is representative of European power market function. However, in reality, markets do not always function perfectly, with an example being in the first quarter of 2017 when several European countries implemented export limits and bans to prevent supply disruptions, which reflected a lack of cooperation in the internal electricity market.[51]

As shown in Figures 1A and 1B, approximate linear relationships are observed between increases in VRE penetration across the scenarios and CO_2 emissions (R^2 = 0.85) and VRE curtailment (R^2 = 0.92). The quality of fit for curtailment reduces to

Table 2. Overview of Simulation Results for Three Scenarios that Represent the Range of Ambition in This Work in Terms of Renewable Energy Penetration

	2015 System	EU Reference 2030	ENTSO-E Vision 3 2030
Wholesale electricity price (€/MWh)	44 (±2.2%)	82 (±2.1%)	60 (±3.6%)
Price received by wind generation (€/MWh)	48 (2.2%)	81 (1.3%)	56 (4.4%)
Price received by solar generation (€/MWh)	45 (2.8%)	86 (1.7%)	40 (4.5%)
Price received by gas generation (€/MWh)	69 (2.5%)	92 (2.0%)	95 (1.8%)
Price received by coal generation (€/MWh)	50 (2.5%)	91 (1.2%)	128 (5.3%)
Price received by nuclear generation (€/MWh)	40 (2.2%)	75 (1.3%)	61 (3.2%)
Total generation cost (€B)	47.11 (±0.8%)	86.83 (±2.1%)	50.28 (±4.2%)
Total CO_2 emissions (Mt)	1001[a] (±1.0%)	917 (±1.3%)	233 (±5.0%)
Emissions intensity (gCO_2/kWh)	322.6 (±1.0%)	247.8 (±1.3%)	68.5 (±5.0%)
RE generation (%)	36.7 (±1.0)	47.2 (±1.4)	68.4 (±1.3)
VRE generation (%)	13.4 (±2.8)	24.4 (±2.7)	35.1 (±2.8)
VRE curtailment (%)	0.1 (±26.3)	0.1 (±16.8)	4.3 (±10.7)
Average interconnection congestion (%)	26.0 (±0.9)	19.1 (±2.6)	29.7 (±1.0)
Total international electricity flow (TWh)	267 (±0.7%)	355 (±2.3%)	411 (±1.2%)

For each metric, the mean and coefficient of variation across all weather years are listed. These scenarios are the 2015 System, the EU Reference, and ENTSO-E vision 3 scenarios (see Supplemental Information for the full range of scenarios). Total generation cost is defined as the sum of total short-run generation costs: fuel, emissions, startup, and shutdown costs. See also Tables S2–S7. RE, renewable energy.
[a]Total electricity emissions from this base year simulation are within 3% of the official verified emissions (1,025 Mt) for this year, using our historical 1985–2014 weather data.[52]

R^2 = 0.79 when the 2015 System simulation is included, suggesting that Europe is expected to begin experiencing notable curtailment due to international constraints beyond a VRE penetration of 22% energy (which is anticipated to be reached by 2027 under conservative EU Reference Scenario conditions[47]). While this simplifies the power system's response by neglecting distribution-level constraints, it provides useful insight into the underlying trends caused by variable renewables and agrees with the broad trajectory from other studies (e.g., the International Energy Agency projects 7% curtailment in 2040[53]).

The year-to-year operational volatility increases with VRE penetration as shown by the 5-fold increase in variability (defined as the inter-annual coefficient of variation) of CO_2 emissions and total generation costs across the scenarios, as shown in Table 2. Due to the reduction in overall CO_2 emissions and increase in VRE penetration, variability of CO_2 emissions increases 5-fold even though the magnitude of CO_2 emissions variability (inter-annual SD) remains broadly consistent across scenarios. This variability in CO_2 emissions implies greater variability in the operation of conventional coal- and gas-fired generation, which generate less with increased variability in their operation. Variability on a country level is greater due to the geographic smoothing of weather systems at a continental level. For example, Great Britain experiences up to 9-fold increase in variability of CO_2 emissions and 7-fold increased variability of total generation costs (see Supplemental Information). Figures 1C and 1D show how the range of wholesale market pricing and total generation costs widens with VRE penetration. Off-model assumptions for fuel and CO_2

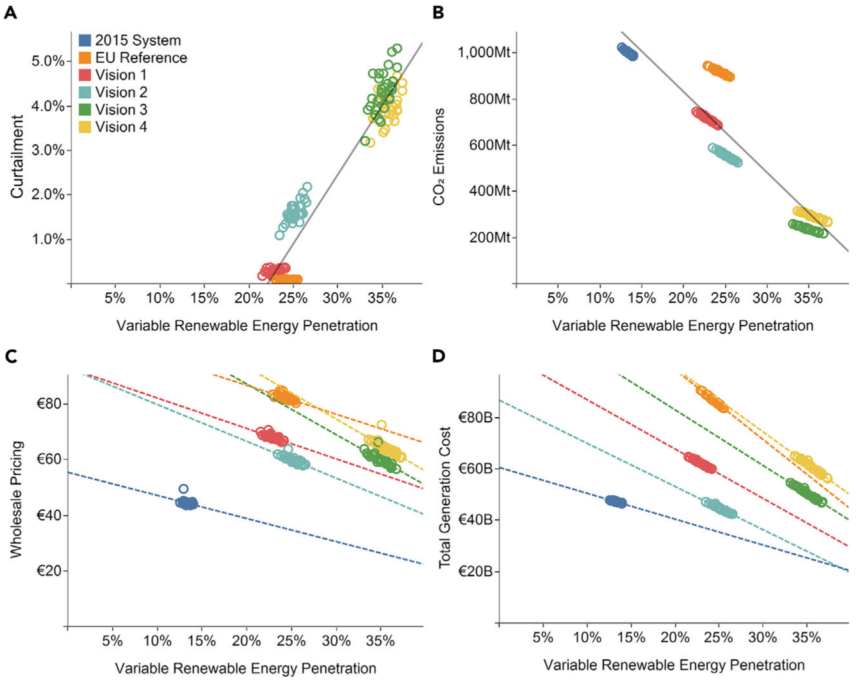

Figure 1. The Relationships between VRE Generation Penetration and Electricity System Metrics Across Historical and 2030 Scenarios

The four panels show (A) VRE curtailment (2015 System simulation excluded), (B) CO_2 emissions, (C) wholesale electricity prices, and (D) total generation cost across all scenarios. Individual points are for individual weather years from the 30-year VRE generation dataset, colors indicate the scenarios. Linear regressions across all scenarios are shown in the top panels, and within individual scenarios in the bottom panels. In (C) and (D), the fitted lines are extrapolated well beyond the range of the data points. They are intended to illustrate the general trend and deliberately do not indicate confidence in the predicted values.

prices strongly influence these outputs, so low correlation is seen across all scenarios between VRE and wholesale prices or total generation costs [$R^2 < 0.1$].

The lines plotted in Figures 1C and 1D show the linear relationships within each scenario, in which only weather inputs change. Total generation costs (Figure 1D) bear strong correlation with average VRE penetration within each scenario [$R^2 = 0.92$], although less so for wholesale market pricing (Figure 1C) [$R^2 = 0.50$]. These lines become steeper with increased penetrations of VRE, indicating that the impact of VRE resource variability on electricity market economics will strengthen and become increasingly volatile with greater penetrations of VRE.

Market Operation and the Displacement of Conventional Fossil-Fueled Generation

With increased VRE penetration and lower fossil generation, carbon price plays a more significant role in determining wholesale electricity prices under the highly decarbonized visions 3 and 4. Fuel prices remain the dominant influence in other scenarios. As shown in Table 2, average wholesale price increases under greater decarbonization, but this increase is not shared equally across all generating technologies. The merit order effect,[5,54,55] whereby VRE depresses prices at times of high output and thus cannibalizes its own revenue, intensifies, especially for solar PV. The price received by solar PV generators decreases relative to 2015 levels. For wind generators, it grows more slowly than the average wholesale price.

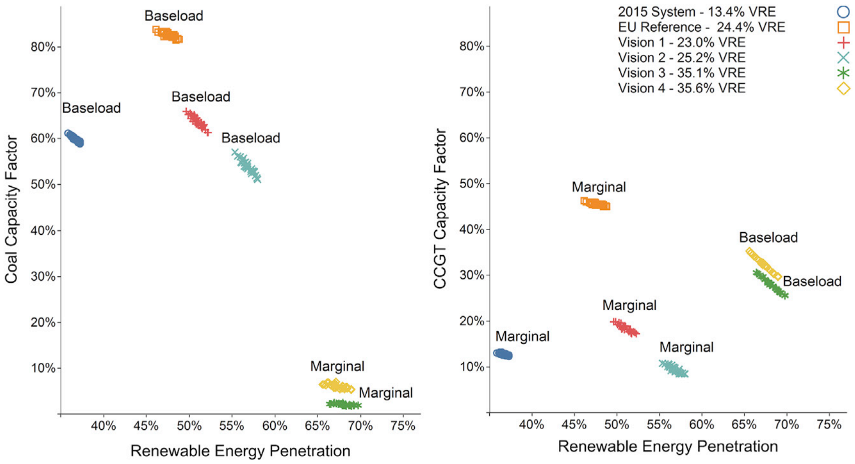

Figure 2. Annual European Coal and Natural Gas Combined Cycle Gas Turbine Capacity Factors by Scenario, Showing the Range across Each of the 30 Historical Weather Years Used

Total renewable energy is defined as VRE plus biomass and hydro power. The labels indicate whether the mode of generation is baseload or marginal in the merit order of each scenario. CCGT, coal and natural gas combined cycle gas turbine.

The price received by fossil-fueled generators increases relative to wholesale prices under decarbonization as their flexibility is more highly valued. However, their utilization is reduced and sees greater year-to-year variability. Fossil-fueled generators account for 63% of power production in the 2015 system scenario, but this falls to just over 30% in renewable energy (RE)>60% scenarios (ENTSO-E visions 3 and 4). This contributes to European emissions intensity falling from an average across weather years of 322 gCO_2/kWh in the 2015 reference scenario to below 100 gCO_2/kWh in those scenarios.

Figure 2 demonstrates that baseload fossil-fired technology (gas in visions 3 and 4, coal otherwise) is most affected by the inter-year variability of VRE because it

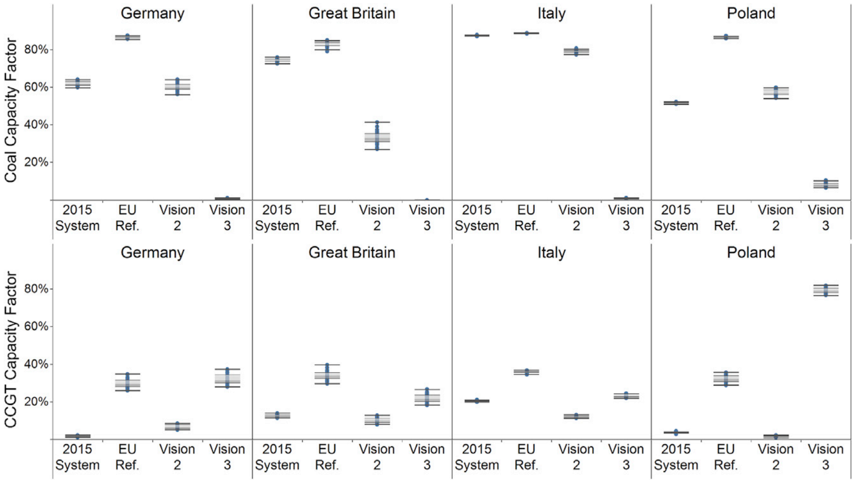

Figure 3. The Range of Capacity Factors for Coal and Natural Gas CCGT Generation Across the 30 Years of Modeled Weather Conditions within Selected Countries

The boxplots show the second and third quartiles in the shaded areas and the whiskers extend to 1.5 times the interquartile range for the selected countries across the 30 years of weather conditions.

CellPress

provides balancing for year-by-year variation in resource availability. Given that Figure 2 depicts the pan-European operation of conventional generators, it masks the more substantial country-level variability. Figure 3 identifies this variability within selected countries and scenarios.

Conventional generators see lower running hours with increased year-to-year variability, implying more challenging financial conditions under energy-only markets. Thus, for these generators to remain financially sustainable, revenues may need to be preserved or given more stability with additional market designs or policies. This may prove pivotal for maintaining security of supply, as these generators mitigate many of the integration challenges associated with increased penetrations of VRE.[56,57] Alternatively, more storage may assist with these challenges, or more transmission coupled with greater heterogeneity in where VRE is located.[32]

Variability of CO_2 Emissions

Increased volatility in the operation of conventional fossil-fueled generation yields a corresponding volatility in CO_2 emissions. Total European CO_2 emissions vary by up to 9% from the long-term average in the RE > 60% scenarios depending on wind and solar resource availability, whether a given year had good or bad weather. In the 2015 system, this difference was 2%. The corresponding Europe-wide maximum variation in VRE power output is around 10% of average total VRE generation for all scenarios considered. With greater penetrations of VRE, the magnitude of this variability increases dramatically. In the 2015 system simulation, it represented 1% of total electricity demand and rose to 4% of total electricity demand in RE > 60% scenarios. Figure 4 illustrates the variability in annual emissions intensity at a country level in both magnitude and as a percentage of average emissions intensity for two scenarios with contrasting ambition, demonstrating that emissions saved by VRE vary substantially depending on the sample year considered. Clearly visible in Figure 4 is that, while the magnitude of emissions variability decreases in many countries, the percentage variability of CO_2 emissions intensity increases across the board.

Figure 5 demonstrates the impact of VRE output on the carbon intensity of electricity generation for selected countries that represent 40% of European electricity demand. Its left side shows the marginal CO_2 emissions intensity reduction from VRE for all scenarios, determined as the gradient of total national emissions intensity against total national percentage share of VRE output over all simulated weather years. This can be interpreted as the reduction in emissions intensity achieved by an increase of one percentage point in VRE penetration. The right-hand portion of Figure 5 displays the emissions intensity of generation for the EU Reference Scenario.

In general, the marginal carbon reduction from renewables decreases as their penetration increases, as the low-hanging fruit (coal) becomes exhausted. Inter-annual variability of emissions intensity also decreases in magnitude with decarbonization ambition but increases as a proportion of overall emissions, as shown in Figure 4. The marginal CO_2 emissions intensity reduction metric yields insights into where decarbonization efforts could be focused to maximize reductions in emissions intensity. The impact of VRE is greatest in Poland (out of the large countries plotted) due to its heavy reliance on coal, thus a one-percentage-point absolute increase in VRE penetration yields a minimum 7 kg/MWh reduction in grid carbon intensity. In contrast, Denmark has much higher VRE penetrations and thus less capability to decarbonize

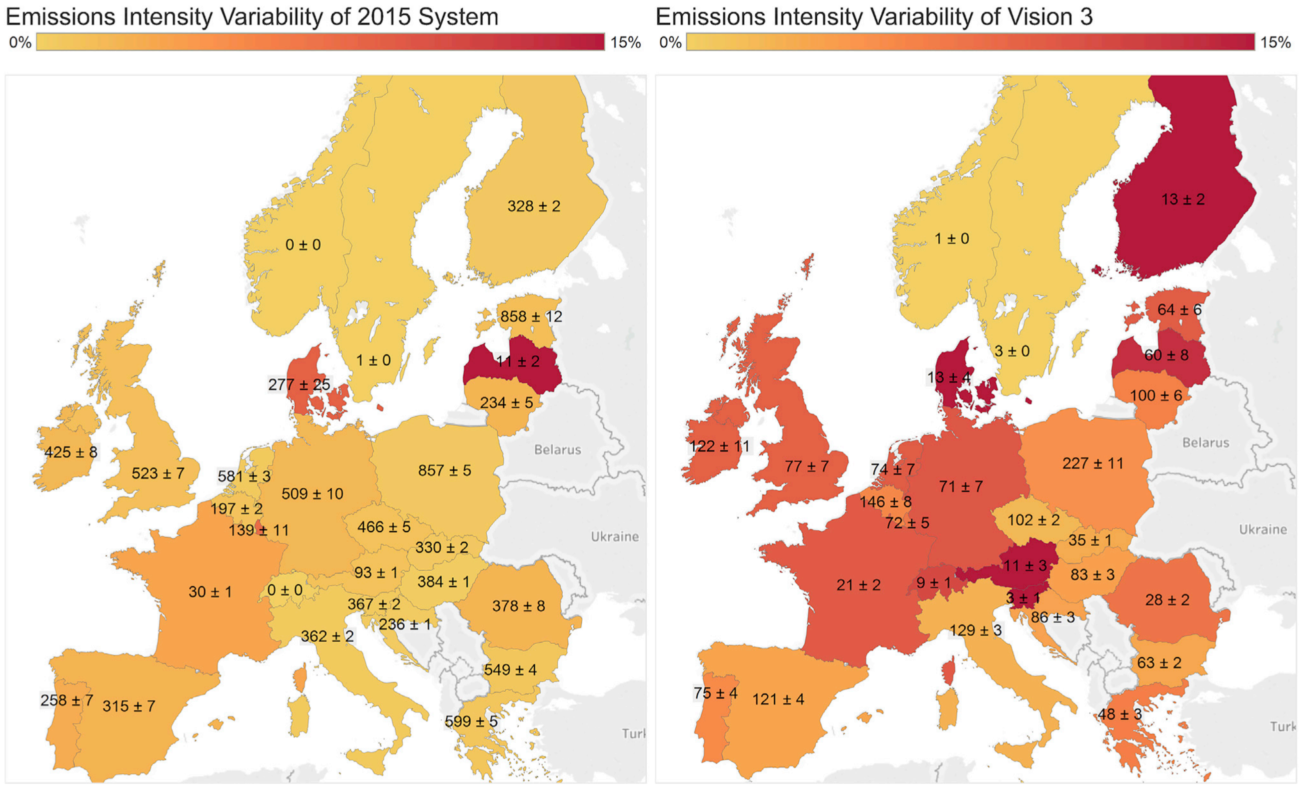

Figure 4. Variability of Electricity CO_2 Emissions Intensity by Country for the 2015 System and Vision 3
For both diagrams, the text on each country describes the mean emissions intensity followed by the standard deviation in kg/MWh over the course of all 30 weather years. The color scale indicates the coefficient of variation for emissions intensity in each country.

further using VRE. This analysis could help guide investments in new VRE capacity to be more efficient at carbon mitigation, and in greater interconnection between countries to limit their reliance on carbon-intensive generation.

The average carbon intensity of electricity decreases marginally during years with higher VRE resource, with $\pm 5\%$ variation from across 30 years averaged over the five countries shown in Figure 5 for the EU Reference Scenario. This inter-annual variability differs strongly between countries due to their generation mix and resulting exposure to VRE variability.

Curtailment of VRE and Interconnector Flows

Curtailment, the limiting of power output, is a method of regulating substantial amounts of VRE power in power systems. Situations that result in curtailment include limited transmission capacity, an oversupply of VRE, and inflexible baseload generation. There is a strong correlation between VRE penetration and curtailment, with near-linear growth above 20% VRE penetration (as shown in Figure 1) and 50% total renewable energy penetration. In our model, curtailment may be caused by operational constraints on generators (minimum stable levels, minimum up and down times), by constraints ensuring demand is met, and by interconnector flow limits between countries. In common with McDonald et al.,[58] we do not consider pumped hydro or battery storage capacity. However, our curtailment levels should still be considered a lower bound, since our model operates under perfect market conditions and does not consider localized network or generation constraints, all of which

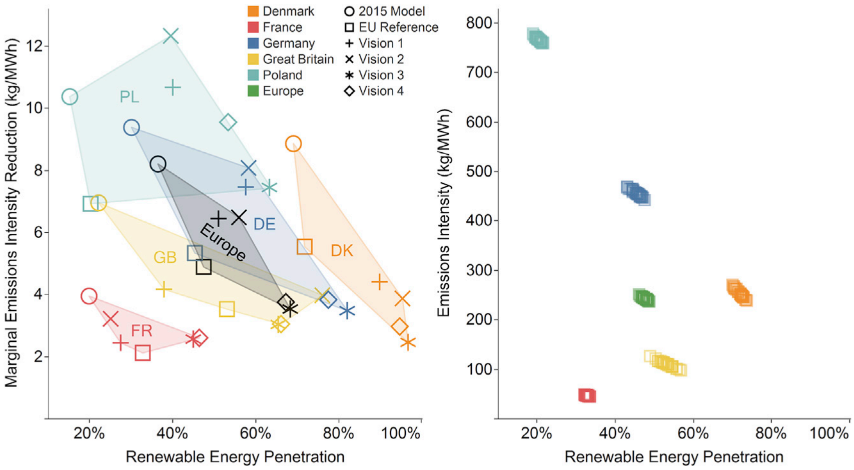

Figure 5. Impact of VRE Output on the Carbon Intensity of Electricity Generation
Marginal reduction in emissions intensity for a 1% increase in VRE penetration for all scenarios averaged across all weather years (left), and average emissions intensity in the EU Reference Scenario for a selection of countries across all weather years (right).

would lead to greater levels of curtailment. For context, Germany and Britain experienced 5%–6% curtailment of wind in 2015, with penetration levels of 12%–13%.[59]

Analyzing curtailment at a European level masks the uneven distribution and interannual variability of curtailment at a country level. Figure 6 presents this country-level variability across weather years. In vision 3, Germany experiences the greatest levels and variability of VRE curtailment, ranging from below 6% to above 10% annually depending on the year, in contrast to the 4.3% $\pm$ 1.2% (51 $\pm$ 15 TWh) at the European level.

While Germany has high levels of curtailment, its neighbor Poland has none. Poland imports substantial amounts of VRE but generates comparatively little. Its resulting carbon-intensive generation (see Figure 5) implies a high marginal emissions intensity reduction potential.

Interconnection is a valuable asset for managing large shares of VRE, with total interconnector flow increasing by up to 80% in RE > 60% scenarios relative to the 2015 system. This increased flow corresponds to greater interdependency between countries and allows an increasingly variable electricity supply to meet demand across broader areas, which smoothens supply-demand mismatches. Interconnector congestion directly restricts the flow of electricity and leads to increased emissions and curtailment of VRE. With targeted infrastructure investment, interconnection capacity could be increased to minimize these factors. As identified in Table 2, interannual flow volatility remains relatively static on interconnector lines and in terms of the overall international flow of electricity. Coupled with a substantial increase in overall interconnector flow, this should continue to provide stable revenues for interconnector operators.

DISCUSSION

Our long-term multi-scenario analysis of European variable renewable power generation maps out for the first time the impacts of long-term weather variability on the operation of a continental power system and how this varies with decarbonization ambition.

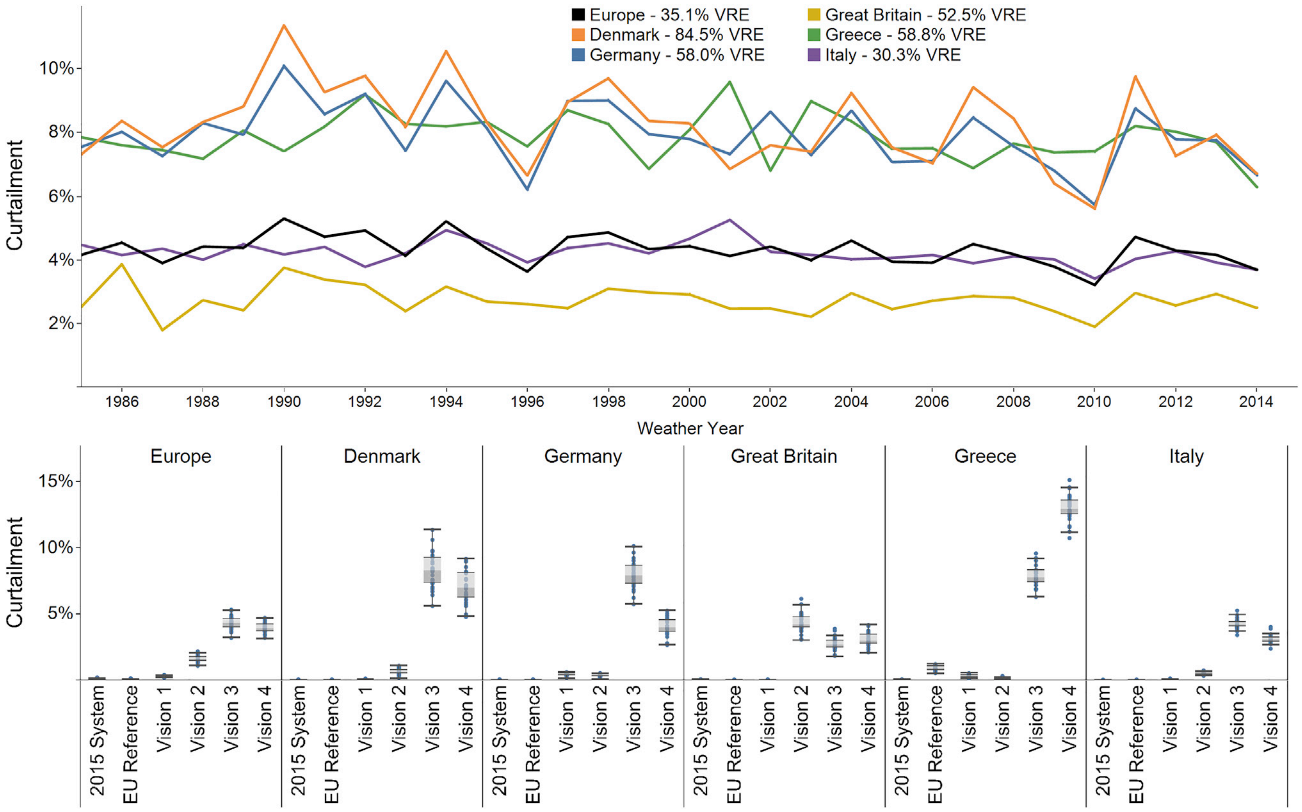

Figure 6. Country-Level Variability of Curtailment of VRE Across Weather Years

The top panel shows selected countries in vision 3 with high levels of curtailment. The bottom panel shows boxplots summarizing these countries within each scenario. The boxplots show the second and third quartiles in the shaded areas and the whiskers extend to 1.5 times the interquartile range for the selected countries across the 30 years of weather conditions.

Increased penetration of weather-dependent renewables leads to increased variability in system operation, with 5-fold growth in the inter-annual variability of CO_2 emissions and total generation costs from the 2015 baseline scenario to the most ambitious 2030 vision. This corresponds to an increased variability in the operation of conventional generators, predominantly those providing baseload, which act to balance out resource availability. Many of these trends can be approximated by simple linear functions of VRE penetration. This allows rapid yet accurate back-of-the-envelope calculations for the impact of renewables deployment in the absence of computationally intensive modeling. Analysis derived from data from a single or small number of years would fail to capture such variability. Thus, estimating decarbonization achievement based on such data is flawed. We find that single-year studies could yield results that deviate by as much as $\pm$9% from the long-term average at a European level and even more at a country level. This also implies that, when measuring progress toward countries' decarbonization targets on a year-by-year basis, weather variability must increasingly be considered as more VRE generation is deployed.

Inevitably, some work must continue to use single-year data due to data availability or computational tractability. Our analysis of three decades of data reveals that the weather years 2012 and 1989 were the most representative for considering power system operation at a European level. This was determined by analyzing the variability of the metrics considered in this paper, which for these years were within $\pm$1%

CellPress

of the 30-year average in relative terms (see Supplemental Information for further information). The years 1990 and 2010 were shown to exhibit the greatest deviation, with our various metrics deviating by ±6% from the long-term average.

A near-doubling of interconnector flow between 2015 and 2030 under ambitious scenarios quantitatively demonstrates an increased interdependency under deep decarbonization of the European power sector. Such interdependency and integrated pan-European operation enable the minimization of operation costs, CO_2 emissions, and variable renewable curtailment. The latter increases linearly beyond 20% penetration of VRE and is an inherent part of a highly variable renewable power system. This should not necessarily be thought of purely as operational inefficiency but rather considered in the context of the costs of additional transmission infrastructure and storage that would be required to make use of curtailed energy. Some curtailment should be acceptable in highly renewable power systems, and the specific level depends on the interplay between the lost value of energy and these additional infrastructure costs. Greater interconnection between countries and the emergence of significant quantities of energy storage (either through dedicated stationary storage or smartly controlled electric vehicle fleets) could facilitate higher shares of renewable energy, as could the emergence of new weather insurance products (e.g., hedging between wind and gas generators to offset revenue risks).

Achieving a decarbonized power system is not without challenges, and this paper maps out a variety of key issues associated with power system decarbonization. However, much remains to be studied and more questions to be asked in order to plan a robust decarbonization of the European power system. For policy developments to be verifiable, interoperable, and representative of the meteorological dependency of decarbonized energy systems, they must be based on open modeling analyses that utilize common long-term datasets, such as those used in this work.[60,61] To this end, we are making our model and all supporting datasets openly available so as to provide the power systems research community with tools to further explore these important issues.

EXPERIMENTAL PROCEDURES

Here we describe the power system scenarios that were considered, the methodologies underpinning the development of the power system dispatch model used, and the wind and solar PV profiles used.

Scenarios Considered

A total of six different power system scenarios were analyzed. The 2015 scenario was developed based on historical electricity demand from ENTSO-E for 2015 and installed capacities based on the European Commission's EU Reference Scenario[47] 2016 results calibrated for the year 2015. The policy scenarios are all for the year 2030, based on the EU Reference Scenario[47] and the ENTSO-E visions.[48] The EU Reference Scenario projects how the European energy system may evolve to 2030 based on business-as-usual assumptions, including full implementation of EU energy and climate policies adopted by December 2014 (for the EU Reference Scenario model, Swiss and Norwegian generation mixes were developed based on ENTSO-E and national strategy documents as they were not part of the EU Reference Scenario[48,62]). The ENTSO-E visions encompass a broad range of possible futures that span a broad range of ambition in terms of the achievement of the sustainability goals within the EU 2050 Roadmap. The four visions provide

the envelope within which the future could plausibly occur but strictly do not act as upper/lower bounds or have a probability of occurrence attached to them.[48] These scenarios informed the electrical load profiles, the efficiency of power generation, and installed generation mix by fuel type in the models constructed.

Modeling Framework

The software used to model the EU electricity market is the PLEXOS Integrated Energy Model,[45] which is widely used for electricity and gas market modeling and planning. In this analysis, the focus is limited to the electricity system; i.e., gas infrastructure and delivery are ignored in these simulations. Within the electricity sector, the model optimizes the dispatch of thermal and renewable generation, holding the installed capacity constant, subject to operational and technical constraints at hourly resolution. The model seeks to minimize the overall generation cost across the EU to meet demand subject to generator technical characteristics such as ramp rates, start costs, and minimum up times. This includes operational costs, consisting of fuel costs and carbon costs, and startup costs, consisting of additional fuel offtake and a fixed unit startup cost. Model equations can be found in.[63] In these simulations, a perfect day-ahead market is assumed across the EU (i.e., no market power or anti-competitive bidding behavior, thus power stations bid their short-run marginal cost) similar to Deane et al.[64]

The models used in this work were developed using a soft-linking approach as in,[46,65,66] whereby the results of long-term analyses are studied using a dedicated power system model to simulate the operational unit commitment and dispatch of the system. Due to the scale of the European power sector and challenges with acquiring granular technical characteristics for the ~10,000 power stations across 30 countries,[67] standard generator classes for 15 modes of generation per node were used with uniform characteristics such as maximum capacities, ramp rates, minimum up and down times, forced outage and maintenance rates, and startup and shutdown costs. All of these technology types have their own standard efficiencies, which themselves differ by country for the years 2015 and 2030 respectively based on values used for these technologies in the EU Reference Scenario for these years. A summary of the main generator characteristics used in this study is available in the Supplemental Information. The resulting market price is defined as the marginal price (note that this is often called the shadow price of electricity) at country level and does not include any extra revenues from potential balancing; reserve or capacity markets; or costs such as grid infrastructure cost, capital costs, or taxes. The models were not constrained for stability issues related to high levels of non-synchronous generation that have been shown to affect the frequency, voltage, and transient and small signal stability of the power system.[56] It was assumed that such operational constraints could be met in ancillary services markets with negligible impact on system operation.

Load Profiles

Each scenario had a unique electrical load profile for each country. For the 2015 system model, historical demand profiles for this year were used as provided by ENTSO-E. For modeling the EU Reference Scenario 2016, the overall energy use was detailed in the results but the profile was not. Thus, it was scaled to 2030 based on the historical hourly 2012 profiles with a peak scaling of 1.1 using PLEXOS, which increased peak load by 10% compared with 2012 levels. For the models of the ENTSO-E four 2030 visions, the hourly load profiles of each scenario were used without the need for adjustment.

Hydro Profiles

Hydro generation is modeled as individual monthly constraints via generation profiles provided by ENTSO-E for each individual Member State of the EU28 and Norway for the year 2012. These monthly constraints are decomposed to hourly profiles in the optimization process.

WIND AND PV PROFILES

We use the Renewables.ninja PV and wind simulation models[49,50] to generate hourly time series of wind and PV generation aggregated to country levels for 30 historical weather years, from 1985 to 2014. The historical weather conditions come from the NASA Modern-Era Retrospective Analysis for Research and Applications, Version 2 (MERRA-2) reanalysis.[68] While satellite irradiance measurements are an alternative source of data for PV simulations,[50] MERRA-2 is used for both PV and wind in order to maintain internal consistency of the dataset and because it exhibits better long-term stability over the three decades considered.

For wind, we extract wind speeds at 2, 10, and 50 m above ground. For PV, global horizontal irradiance and direct normal irradiance are estimated from surface and top-of-atmosphere incident shortwave flux variables. Surface temperature is used to compute temperature-dependent panel efficiency. We model individual wind farms ($\sim$10,000 across Europe), considering the specific location and characteristics of each farm (turbine model and hub height). Missing data are inferred using multivariate regression (e.g., if the hub height of a particular farm is not known it will be inferred based on the turbine capacity, year of installation, and the country it is located in).

There is no consistent and accurate spatially resolved dataset for all existing European PV installations. For PV, we therefore simulate an installation in each MERRA-2 grid cell (assigning these cells to countries and with each country scaled to its installed capacity). We assume probabilistic panel alignment and inclination, sampled from normal distributions fitted to observed panels installed across Europe.[50] We modeled azimuth as $180^\circ \pm 40^\circ$ (clipped to [0, 360]), and tilt as latitude $\pm 15^\circ$ (clipped to [0, 90]).

For each of the four visions, solar power is scaled to the national totals accordingly, while the wind fleet is based on the commercial planning pipeline currently in place. Existing farms are assumed to all still be in existence, then new farms are added until the capacity specified by the scenario is reached. Capacity is added by first drawing randomly from farms under construction, then those with approved planning permission, and finally those earlier on in the planning pipeline. For these planned future wind farms, the anticipated hub height, technology, and location are accounted for.[49] Thus, the future time series of wind output account for anticipated technological progress out to 2030.

SUPPLEMENTAL INFORMATION

Supplemental Information includes Supplemental Experimental Procedures, five figures, and eight tables and can be found with this article online at https://doi.org/10.1016/j.joule.2018.06.020.

ACKNOWLEDGMENTS

S.C., P.D., and B.Ó.G. were supported by the Science Foundation Ireland (SFI) MaREI Centre (12/RC/2302). S.P. was supported by the European Research Council via grant StG 2012-313553. I.S. was supported by EPSRC project EP/M001369/1.

AUTHOR CONTRIBUTIONS

S.C. developed the power system model and wrote the first draft of the paper. P.D. validated the power system model. S.P. and I.S. developed the hourly time series of PV and wind generation aggregated to country levels for 30 historical weather years used within the power system model. B.Ó G. provided guidance and reviewed drafts. All authors contributed to designing the research, analyzing the results, and refining the paper.

DECLARATION OF INTERESTS

The authors declare no competing interests.

Received: April 24, 2018
Revised: June 7, 2018
Accepted: June 28, 2018
Published: July 26, 2018

REFERENCES

1. IRENA (2017). Renewable Electricity Capacity and Generation Statistics. http://www.irena.org/publications/2017/Jul/Renewable-Energy-Statistics-2017.
2. Bird, L., Lew, D., Milligan, M., Carlini, E.M., Estanqueiro, A., Flynn, D., Gomez-Lazaro, E., Holttinen, H., Menemenlis, N., Orths, A., et al. (2016). Wind and solar energy curtailment: a review of international experience. Renew. Sustain. Energy Rev. *65*, 577–586.
3. Pean, E., Pirouti, M., and Qadrdan, M. (2016). Role of the GB-France electricity interconnectors in integration of variable renewable generation. Renew. Energy *99*, 307–314.
4. Higgins, P., Li, K., Devlin, J., and Foley, A.M. (2015). The significance of interconnector counter-trading in a security constrained electricity market. Energy Policy *87*, 110–124.
5. Sensfuß, F., Ragwitz, M., and Genoese, M. (2008). The merit-order effect: a detailed analysis of the price effect of renewable electricity generation on spot market prices in Germany. Energy Policy *36*, 3086–3094.
6. Würzburg, K., Labandeira, X., and Linares, P. (2013). Renewable generation and electricity prices: taking stock and new evidence for Germany and Austria. Energy Econ. *40* (*Suppl. 1*), S159–S171.
7. Winkler, J., Pudlik, M., Ragwitz, M., and Pfluger, B. (2016). The market value of renewable electricity – which factors really matter? Appl. Energy *184*, 464–481.
8. Poncelet, K., Delarue, E., Six, D., Duerinck, J., and D'haeseleer, W. (2016). Impact of the level of temporal and operational detail in energy-system planning models. Appl. Energy *162*, 631–643.
9. Pietzcker, R.C., Ueckerdt, F., Carrara, S., De Boer, H.-S., Després, J., Fujimori, S., Johnson, N., Kitous, A., Scholz, Y., Sullivan, P., et al. (2017). System integration of wind and solar power in Integrated Assessment Models: a cross-model evaluation of new approaches. Energy Econ. *64*, 583–599.
10. Lu, X., McElroy, M.B., and Kiviluoma, J. (2009). Global potential for wind-generated electricity. Proc. Natl. Acad. Sci. USA *106*, 10933–10938.
11. Schroeder, A., Oei, P.-Y., Sander, A., Hankel, L., and Laurisch, L.C. (2013). The integration of renewable energies into the German transmission grid—a scenario comparison. Energy Policy *61*, 140–150.
12. Pfenninger, S., and Keirstead, J. (2015). Renewables, nuclear, or fossil fuels? Scenarios for Great Britain's power system considering costs, emissions and energy security. Appl. Energy *152*, 83–93.
13. Rodriguez, R.A., Becker, S., and Greiner, M. (2015). Cost-optimal design of a simplified, highly renewable pan-European electricity system. Energy *83*, 658–668.
14. Widen, J. (2011). Correlations between large-scale solar and wind power in a future scenario for Sweden. IEEE Trans. Sustain. Energy *2*, 177–184.
15. Drew, D.R., Cannon, D.J., Brayshaw, D.J., Barlow, J.F., and Coker, P.J. (2015). The impact of future offshore wind farms on wind power generation in Great Britain. Resources *4*, 155–171.
16. Andresen, G.B., Søndergaard, A.A., and Greiner, M. (2015). Validation of Danish wind time series from a new global renewable energy atlas for energy system analysis. Energy *93*, 1074–1088.
17. Olauson, J., and Bergkvist, M. (2015). Modelling the Swedish wind power production using MERRA reanalysis data. Renew. Energy *76*, 717–725.
18. Staffell, I. (2014). Developing an Open-Access Wind Profile for Great Britain (Department of Energy & Climate Change), p. 1025.
19. Staffell, I., and Green, R. (2014). How does wind farm performance decline with age? Renew. Energy *66*, 775–786.
20. Bonjean Stanton, M.C., Dessai, S., and Paavola, J. (2016). A systematic review of the impacts of climate variability and change on electricity systems in Europe. Energy *109*, 1148–1159.
21. Schaeffer, R., Szklo, A.S., Pereira de Lucena, A.F., Moreira Cesar Borba, B.S., Pupo Nogueira, L.P., Fleming, F.P., Troccoli, A., Harrison, M., and Boulahya, M.S. (2012). Energy sector vulnerability to climate change: a review. Energy *38*, 1–12.
22. Klein, D.R., Olonscheck, M., Walther, C., and Kropp, J.P. (2013). Susceptibility of the European electricity sector to climate change. Energy *59*, 183–193.
23. Chandramowli, S.N., and Felder, F.A. (2014). Impact of climate change on electricity systems and markets – a review of models and forecasts. Sustain. Energy Technol. Assess. *5*, 62–74.
24. Pfenninger, S., Hawkes, A., and Keirstead, J. (2014). Energy systems modeling for twenty-first century energy challenges. Renew. Sustain. Energy Rev. *33*, 74–86.
25. Collins, S., Deane, J.P., Poncelet, K., Panos, E., Pietzcker, R.C., Delarue, E., and Ó Gallachóir, Brian P. (2017). Integrating short term variations of the power system into integrated energy system models: a methodological review. Renew. Sustain. Energy Rev. *76*, 839–856.
26. IRENA (2017). Planning for the renewable future: long-term modelling and tools to expand variable renewable power in emerging economies. http://www.irena.org/publications/2017/Jan/Planning-for-the-renewable-future-Long-term-modelling-and-tools-to-expand-variable-renewable-power.
27. Pfenninger, S. (2017). Dealing with multiple decades of hourly wind and PV time series in energy models: a comparison of methods to reduce time resolution and the planning implications of inter-annual variability. Appl. Energy *197*, 1–13.
28. Bloomfield, H., Brayshaw, D.J., Shaffrey, L.C., Coker, P.J., and Thornton, H. (2016). Quantifying the increasing sensitivity of power systems to climate variability. Environ. Res. Lett. *11*, 124025.

29. Shaner, M.R., Davis, S.J., Lewis, N.S., and Caldeira, K. (2018). Geophysical constraints on the reliability of solar and wind power in the United States. Energy Environ. Sci. *11*, 914–925.

30. Olauson, J., Ayob, M.N., Bergkvist, M., Carpman, N., Castellucci, V., Goude, A., Lingfors, D., Waters, R., and Widén, J. (2016). Net load variability in Nordic countries with a highly or fully renewable power system. Nat. Energy *1*, 16175.

31. Burtin, A., and Silva, V. (2015). Technical and economic analysis of the European electricity system with 60% RES. http://www.energypost.eu/wp-content/uploads/2015/06/EDF-study-for-download-on-EP.pdf.

32. Grams, C.M., Beerli, R., Pfenninger, S., Staffell, I., Wernli, H., Schmaljohann, H., Both, C., Masnadi, M.S., Brandt, A.R., and Spence, P. (2017). Balancing Europe's wind-power output through spatial deployment informed by weather regimes AOP. Perspective. https://www.nature.com/articles/nclimate3338.

33. Jacobson, M.Z., Delucchi, M.A., Cameron, M.A., and Frew, B.A. (2017). The United States can keep the grid stable at low cost with 100% clean, renewable energy in all sectors despite inaccurate claims. Proc. Natl. Acad. Sci. USA *114*, E5021–E5023.

34. Clack, C.T.M., Qvist, S.A., Apt, J., Bazilian, M., Brandt, A.R., Caldeira, K., Davis, S.J., Diakov, V., Handschy, M.A., Hines, P.D.H., et al. (2017). Evaluation of a proposal for reliable low-cost grid power with 100% wind, water, and solar. Proc. Natl. Acad. Sci. USA *114*, 6722–6727.

35. Porter, E. (2017). Fisticuffs over the route to a clean-energy future. New York Times. https://www.nytimes.com/2017/06/20/business/energy-environment/renewable-energy-national-academy-matt-jacobson.html.

36. Editorial. (2017). Business as unusual. Nat. Energy *2*, 17150.

37. Fosso, O.B., and Belsnes, M.M. (2004). Short-term hydro scheduling in a liberalized power system. In Power System Technology, 2004. PowerCon 2004 (IEEE). https://doi.org/10.1109/ICPST.2004.1460206.

38. Hyndman, R.J., and Fan, S. (2010). Density forecasting for long-term peak electricity demand. IEEE Trans. Power Syst. *25*, 1142–1153.

39. Hdidouan, D., and Staffell, I. (2017). The impact of climate change on the levelised cost of wind energy. Renew. Energy *101*, 575–592.

40. Jerez, S., Tobin, I., Vautard, R., Montávez, J.P., López-Romero, J.M., Thais, F., Bartok, B., Christensen, O.B., Colette, A., Déqué, M., et al. (2015). The impact of climate change on photovoltaic power generation in Europe. Nat. Commun. *6*, 10014.

41. Crook, J.A., Jones, L.A., Forster, P.M., and Crook, R. (2011). Climate change impacts on future photovoltaic and concentrated solar power energy output. Energy Environ. Sci. *4*, 3101–3109.

42. Pryor, S.C., and Barthelmie, R.J. (2010). Climate change impacts on wind energy: a review. Renew. Sustain. Energy Rev. *14*, 430–437.

43. Wohland, J., Reyers, M., Weber, J., and Witthaut, D. (2017). More homogeneous wind conditions under strong climate change decrease the potential for inter-state balancing of electricity in Europe. Earth Syst. Dynam. *8*, 1047–1060.

44. Kovats, R.S., Valentini, R., Bouwer, L.M., Georgopoulou, E., Jacob, D., Martin, E., Rounsevell, M., and Soussana, J.F. (2014). 2014: Europe. In Climate Change 2014: Impacts, Adaptation, and Vulnerability. Part B: Regional Aspects. Contribution of Working Group II to the Fifth Assessment Report of the Intergovernmental Panel on Climate Change, V.R. Barros, C.B. Field, D.J. Dokken, M.D. Mastrandrea, K.J. Mach, T.E. Bilir, M. Chatterjee, K.L. Ebi, Y.O. Estrada, and R.C. Genova, et al., eds. (Cambridge University Press), pp. 1267–1326.

45. Energy-Exemplar. (2016). PLEXOS Integrated Energy Model. http://energyexemplar.com/software/plexos-desktop-edition/.

46. Deane, J.P., Chiodi, A., Gargiulo, M., and Ó Gallachóir, B.P. (2012). Soft-linking of a power systems model to an energy systems model. Energy *42*, 303–312.

47. EC (2016). EU reference scenario 2016. Energy, transport and GHG emissions. Trends to 2050. Main results. https://ec.europa.eu/energy/en/data-analysis/energy-modelling.

48. ENTSOE (2016). TYNDP 2016 Scenario development report. https://docstore.entsoe.eu/Documents/TYNDP%20documents/TYNDP%202016/150521_TYNDP2016_Scenario_Development_Report_for_consultationv2.pdf.

49. Staffell, I., and Pfenninger, S. (2016). Using bias-corrected reanalysis to simulate current and future wind power output. Energy *114*, 1224–1239.

50. Pfenninger, S., and Staffell, I. (2016). Long-term patterns of European PV output using 30 years of validated hourly reanalysis and satellite data. Energy *114*, 1251–1265.

51. EC (2017). Quarterly report on European electricity markets, volume 10 (issue 1; first quarter of 2017). https://ec.europa.eu/energy/en/data-analysis/market-analysis.

52. EEA. (2016). Trends and projections in the EU ETS in 2016–The EU Emissions Trading System in numbers. Report No 24/2016. https://www.eea.europa.eu/publications/trends-and-projections-EU-ETS-2016.

53. IEA (2017). Digitalization & energy. http://www.iea.org/publications/freepublications/publication/DigitalizationandEnergy3.pdf.

54. Hirth, L. (2013). The market value of variable renewables: the effect of solar wind power variability on their relative price. Energy Econ. *38*, 218–236.

55. Staffell, I. (2017). Measuring the progress and impacts of decarbonising British electricity. Energy Policy *102*, 463–475.

56. Flynn, D., Rather, Z., Ardal, A., D'Arco, S., Hansen, A.D., Cutululis, N.A., Sorensen, P., Estanquiero, A., Gómez, E., Menemenlis, N., et al. (2017). Technical impacts of high penetration levels of wind power on power system stability. Wiley Interdiscip. Rev. Energy Environ. *6*, e216.

57. EirGrid (2017). Operational Constraints Update 15th March 2017 (EirGrid).

58. MacDonald, A.E., Clack, C.T.M., Alexander, A., Dunbar, A., Wilczak, J., and Xie, Y. (2016). Future cost-competitive electricity systems and their impact on US CO_2 emissions. Nat. Clim. Chang. *6*, 526.

59. Joos, M., and Staffell, I. (2018). Short-term integration costs of variable renewable energy: wind curtailment and balancing in Britain and Germany. Renew. Sustain. Energy Rev. *86*, 45–65.

60. Pfenninger, S., DeCarolis, J., Hirth, L., Quoilin, S., and Staffell, I. (2017). The importance of open data and software: is energy research lagging behind? Energy Policy *101*, 211–215.

61. Pfenninger, S. (2017). Energy scientists must show their workings. Nature *542*, 393.

62. Stappel, M., Gerlach, A.-K., Scholz, A., and Pape, C. (2015). The European Power System in 2030: Flexibility Challenges and Integration Benefits (Agora Energiewende). https://www.agora-energiewende.de/fileadmin/Projekte/2014/Ein-flexibler-Strommarkt-2030/Agora_European_Flexibility_Challenges_Integration_Benefits_WEB_Rev1.pdf.

63. Deane, J.P., Drayton, G., and Ó Gallachóir, B.P. (2014). The impact of sub-hourly modelling in power systems with significant levels of renewable generation. Appl. Energy *113*, 152–158.

64. Deane, J., Driscoll, Á., and Ó Gallachóir, B. (2015). Quantifying the impacts of national renewable electricity ambitions using a North–West European electricity market model. Renew. Energy *80*, 604–609.

65. Deane, J., Gracceva, F., Chiodi, A., Gargiulo, M., and Ó Gallachóir, B. (2015). Soft-linking exercises between TIMES, power system models and housing stock models. In Informing Energy and Climate Policies Using Energy Systems Models, G. Giannakidis, M. Labriet, B. Ó Gallachóir, and G. Tosato, eds. (Springer), pp. 315–331.

66. Deane, J., Gracceva, F., Chiodi, A., Gargiulo, M., and Gallachóir, B.P. (2015). Assessing power system security. A framework and a multi model approach. Int. J. Electr. Power Energy Syst. *73*, 283–297.

67. Green, R., and Staffell, I. (2016). Electricity in Europe: exiting fossil fuels? Oxf. Rev. Econ. Pol. *32*, 282–303.

68. Gelaro, R., McCarty, W., Suárez, M.J., Todling, R., Molod, A., Takacs, L., Randles, C.A., Darmenov, A., Bosilovich, M.G., Reichle, R., et al. (2017). The modern-era retrospective analysis for research and applications, version 2 (MERRA-2). J. Clim. *30*, 5419–5454.

JOUL, Volume *2*

Supplemental Information

Impacts of Inter-annual Wind and Solar Variations on the European Power System

Seán Collins, Paul Deane, Brian Ó Gallachóir, Stefan Pfenninger, and Iain Staffell

This document is Creative Commons Attribution 4.0 licensed.

1. AVAILABILITY OF MODELS AND DATA

The PLEXOS model used in this study is available at:

https://energyexemplar.com/datasets/

The Renewables.ninja PV and wind generation dataset is available at:

https://www.renewables.ninja/downloads

2. MODELS AND THEIR ASSUMPTIONS

While all modelling assumptions and data sources underpinning this work have been provided in the manuscript, this section serves to more thoroughly detail the models used to provide further information for the reader regarding the underlying assumptions and implicit limitations of this study.

2.1. PLEXOS Integrated Energy Model

PLEXOS Integrated Energy Model is a power system modelling platform developed by Energy Exemplar that is used for integrated modelling of power, gas and water systems[1]. It is a commercial modelling tool that is free of charge for non-commercial research applications in academic institutions.

In this study, the modelling platform is used to optimise unit commitment and economic dispatch of the power sector by using short term deterministic modelling. The model developed assumes perfect foresight in relation to electrical load, wind and solar production, and forced outages of generators. Stochastic unit commitment is possible within PLEXOS and could be applied to represent imperfect foresight in relation to these parameters. However, this was not applied in this work due to it being outside the scope given the disparity between its typical application in shorter term dispatch planning and our 30 year long period of analysis. This means that while the work is representative of day ahead market modelling, it is idealised and does not represent the imperfect reality of shorter term market function. A perfect day-ahead market is assumed across the EU where there is no market power or anti-competitive bidding behaviour where power stations bid their

true short-run marginal cost, all of which impact the reality of power system operation.

While PLEXOS can be used for optimising investment, this function was not used in this work and only a dispatch simulation of six discreet scenarios were undertaken. Operations planning models, by definition, are not best suited to assessing optimal investment but this work shows how they leveraged to gain insights into the weather induced variability of system operation.

The model minimises the total generation cost of the system while respecting four key constraints: 1) electricity demand and supply must balance; 2) technical characteristics of generators (such as minimum stable levels, ramp rates, minimum up and down times, and maintenance rates); 3) transmission capacity of interconnector lines; 4) forced (random outages based on Monte Carlo simulations) and unforced (scheduled) outages of generators.

PLEXOS models each trading period and ensures chronological consistency across the entire horizon of the optimisation. It models the start-up and shutdown of all generators and tracks them over time. It models the unit commitment and economic dispatch of the power system over the entire time horizon. To ensure consistency across the entire optimisation horizon and avoid problems with inter-temporal constraints at simulation boundary steps, a look ahead period is used which gives the optimiser information about what happens after the period of optimisation. With this look ahead period, the optimiser solves the simulation period and the look ahead period in combination but only keeps the results for the simulation period. In the PLEXOS modelling framework, hydro, wind, solar PV and other renewables are typically treated as free modes of generation with zero marginal cost. This is how they were treated in this study and led them to be dispatched as much as possible subject to resource availability so as to minimise total generation costs.

As mentioned in the manuscript, a core challenge in this work surrounded the acquisition of granular technical characteristic for the ~10,000 power stations across 30 European countries. This led the use of standard generator classes for 15 modes of generation for each country with homogenous characteristics. A summary of the key generator characteristics used in this study is provided below in Table S1. Of course, use of these standard characteristics and standard fuel pricing across Europe will impact results for each scenario. Model results, however, are very sensitive to such differences in generator characteristics and fuel pricing, and unless a comprehensive reliable data source is available, their application would lead to bias in the model results.

Table S1: Standard generator characteristics used in PLEXOS model

Fuel Type	Capacity (MW)	Start Cost (€)	Minimum Stable Generation	Ramp Rate (MW/min)
Biomass-waste fired	300	10,000	30%	30
Derived gasses	150	12,000	40%	30
Geothermal heat	70	3,000	40%	30
Hydro Lakes	150	0	0%	30
Hydro Run of River	200	0	0%	30
Hydrogen plants	300	5,000	40%	30
Natural gas CCGT	450	80,000	40%	30
Natural gas OCGT	100	10,000	20%	30
Nuclear energy	1200	120,000	60%	30
Oil fired	400	75,000	40%	30
Solids fired	300	80,000	30%	30

The model was simulated using the MOSEK solver with rounded relaxation unit commitment, a duality gap of 0.05% and a six hour look ahead. In line with the EU Target Model day-ahead market-scheduling algorithm, known as EUPHEMIA[2], 365 days of the each scenario simulation year were simulated at hourly resolution.

2.2. Renewables.ninja wind and PV generation datasets

The Renewables.ninja PV and wind simulation models[3; 4] were used to generate hourly time series of wind and PV generation aggregated to country levels for 30 historical weather years, from 1985 to 2014.

Renewables.ninja uses the NASA MERRA-2 global meteorological reanalysis[5] to provide consistent weather input data for wind and PV generation. As discussed in Refs.[3; 4] MERRA-2 has many advantages over other global reanalyses, in particular, it provides observations at hourly intervals and has a high spatial resolution of 0.5° latitude and 0.625° longitude[6]. Reanalysis data are known to require bias correction due to systemic errors in the assimilation of data through the underlying weather model, their spatial coarseness and their representation of wind speeds at actual wind farm sites[7]. The Renewables.ninja data are bias-corrected by validation with historic solar PV and wind generation as described in Refs.[3; 4].

Renewables.ninja uses the Global Solar Energy Estimator (GSEE) model[3] for solar PV and the Virtual Wind Farm (VWF) model[8] for wind generation. GSEE was used to simulate PV power output from panels with probabilistic tilt and azimuth angles

drawn from a distribution of known panel angles in Europe, in each MERRA-2 grid cell, the results of which are then aggregated to country level data. The VWF model was used to simulate specific individual wind farms in Europe, both existing and planned, the results of which are aggregated to country level. This is not possible for PV systems due to lacking information about distributed PV installations across Europe.

The resulting bias-corrected datasets show good agreement with reported aggregated generation data (see Refs.[3; 4]). Future work on simulating Europe's decarbonised power system at higher spatial resolutions than the country-aggregated level used here will nevertheless benefit from using newer reanalyses with higher spatial resolution, regional reanalyses, or other more highly resolved datasets such as direct satellite-measured data.

3. EXTENDED RESULTS

3.1. Results Overview: Europe

Table S2: Results overview for Europe

			ENTSOE			
	2015 System	EU Reference 2030	Vision 1 2030	Vision 2 2030	Vision 3 2030	Vision 4 2030
Electricity Price[1] (€/MWh)	44 (32.2%)	82 (32.1%)	68 (31.4%)	60 (32.1%)	60 (33.6%)	64 (33.7%)
Wind-weighted Price (€/MWh)[2]	48 (2.2%)	81 (1.3%)	68 (1.8%)	58 (3.2%)	56 (4.4%)	63 (4.2%)
Solar-weighted Price (€/MWh)	45 (2.8%)	86 (1.7%)	66 (1.9%)	54 (2.6%)	40 (4.5%)	39 (5.7%)
Gas-weighted Price (€/MWh)	69 (2.5%)	92 (2.0%)	96 (1.4%)	105 (2.2%)	95 (1.8%)	99 (2.0%)
Coal-weighed Price (€/MWh)	50 (2.5%)	91 (1.2%)	77 (1.1%)	75 (1.2%)	128 (5.3%)	124 (3.7%)
Nuclear-weighted Price (€/MWh)	40 (2.2%)	75 (1.3%)	61 (1.3%)	54 (2.1%)	61 (3.2%)	66 (3.7%)
Total Generation Cost[3] (€ bn)	47.11 (30.8%)	86.83 (32.1%)	62.09 (32.1%)	44.39 (33.0%)	50.28 (34.2%)	60.47 (34.0%)
Total CO_2 Emissions (Mt)	1001 (31.0%)[4]	917 (31.3%)	713 (32.1%)	551 (33.0%)	233 (35.0%)	288 (34.7%)
Carbon Intensity (gCO_2/kWh)	322.6 (31.0%)	247.8 (31.3%)	209.7 (32.1%)	167.0 (33.0%)	68.5 (35.0%)	80.0 (34.7%)
RE Generation Share	36.7% (31.0%)	47.2% (31.4%)	51.0% (31.3%)	57.0% (31.3%)	68.4% (31.3%)	67.4% (31.3%)
VRE Generation Share	13.4% (32.8%)	24.4% (32.7%)	23.0% (32.9%)	25.2% (33.0%)	35.1% (32.8%)	35.6% (32.7%)
VRE Curtailment	0.1% (326.3%)	0.1% (316.8%)	0.3% (318.5%)	1.6% (314.5%)	4.3% (310.7%)	4% (38.8%)
Interconnector Congestion[5]	26.0% (30.9%)	19.1% (32.6%)	25.1% (32.2%)	28.3% (31.9%)	29.7% (31.0%)	35.0% (30.8%)
Total International Electricity Flow	267 TWh (30.7%)	355 TWh (32.3%)	441 TWh (31.5%)	454 TWh (31.6%)	411 TWh (31.2%)	480 TWh (30.9%)

[1] Wholesale electricity price is defined as the marginal cost of electricity in each region, reflecting the shadow price on the electricity demand-supply constraint. This captures an uplift element to account for start-up costs of thermal plant but excludes taxes, capacity payments or ancillary services. This should be interpreted as an energy-only price in a perfect wholesale market where no market power of strategic behaviours occurs.
[2] Average price received by wind generators (also referred to as 'capture price')
[3] Total Generation Cost = Generation Cost + Start & Shutdown Cost + Emissions Cost
[4] Total electricity emissions from this base year simulation is within 3% of the official verified emissions (1025 Mt) for this year, using our historical 1985-2014 weather data.
[5] Averaged over all transmission lines

3.2. Results Overview: Germany

Table S3: Results overview for Germany

	2015 System	EU Reference 2030	ENTSOE Vision 1 2030	Vision 2 2030	Vision 3 2030	Vision 4 2030
Electricity Price (€/MWh)	46 (36.5%)	105 (31.9%)	78 (32.5%)	73 (32.3%)	59 (35.1%)	68 (34.6%)
Wind-weighted Price (€/MWh)	41 (6.0%)	95 (2.3%)	67 (3.7%)	64 (3.5%)	45 (7.0%)	53 (6.0%)
Solar-weighted Price (€/MWh)	39 (7.8%)	114 (2.8%)	70 (3.3%)	58 (3.9%)	39 (5.7%)	45 (6.9%)
Gas-weighted Price (€/MWh)	99 (48.8%)	115 (3.2%)	105 (5.6%)	101 (4.3%)	89 (2.5%)	93 (2.2%)
Coal-weighed Price (€/MWh)	47 (5.7%)	104 (1.9%)	83 (1.7%)	79 (1.5%)	107 (23.3%)	118 (14.4%)
Nuclear-weighted Price (€/MWh)	45 (6.2%)	0 (0%)	0 (0%)	0 (0%)	0 (0%)	0 (0%)
Total Generation Cost (€ bn)	9.16 (31.7%)	19.70 (32.2%)	12.61 (33.0%)	10.77 (33.2%)	7.66 (37.8%)	10.01 (36.8%)
Total CO_2 Emissions (Mt)	270 (31.9%)	288 (31.0%)	221 (32.7%)	190 (33.3%)	38 (38.6%)	50 (37.8%)
Carbon Intensity (gCO_2/kWh)	509.1 (31.9%)	455.5 (31.5%)	372.0 (33.3%)	369.3 (33.5%)	70.7 (39.6%)	91.1 (38.7%)
RE Generation Share	30.2% (33.3%)	45.4% (32.6%)	57.6% (32.6%)	58.5% (32.7%)	82.2% (32.0%)	77.6% (32.4%)
VRE Generation Share	22.7% (34.4%)	36.8% (33.4%)	41.2% (33.9%)	37.2% (34.3%)	58.0% (33.3%)	56.5% (33.6%)
VRE Curtailment	0.0% (30%)	0.0% (30%)	0.4% (327.4%)	0.3% (328.7%)	7.9% (312.4%)	7.9% (38.2%)
Interconnector Congestion	25.1% (31.8%)	16.4% (34.2%)	24.4% (34.0%)	22.3% (33.6%)	31.7% (32.1%)	32.6% (31.9%)
Total International Electricity Flow	53 TWh (31.6%)	79 TWh (33.6%)	105 TWh (33.0%)	100 TWh (32.7%)	109 TWh (31.9%)	121 TWh (31.6%)

3.3. Results Overview: Spain

Table S4 - Results overview for Spain

	2015 System	EU Reference 2030	ENTSOE Vision 1 2030	ENTSOE Vision 2 2030	ENTSOE Vision 3 2030	ENTSOE Vision 4 2030
Electricity Price (€/MWh)	52 (31.9%)	81 (1.4%)	76 (31.5%)	68 (32.3%)	71 (33.5%)	68 (34.9%)
Wind-weighted Price (€/MWh)	47 (2.7%)	76 (1.6%)	69 (1.6%)	63 (2.9%)	64 (3.8%)	63 (5.1%)
Solar-weighted Price (€/MWh)	49 (1.6%)	72 (1.4%)	64 (1.8%)	43 (3.3%)	48 (4.4%)	30 (6.4%)
Gas-weighted Price (€/MWh)	57 (1.9%)	88 (2.6%)	95 (2.7%)	100 (3.4%)	85 (2.7%)	91 (3.9%)
Coal-weighed Price (€/MWh)	52 (1.9%)	83 (1.1%)	79 (1.3%)	79 (1.8%)	92 (27.2%)	113 (24.5%)
Nuclear-weighted Price (€/MWh)	51 (1.8%)	81 (1.3%)	75 (1.3%)	69 (2.4%)	72 (3.5%)	73 (4.8%)
Total Generation Cost (€ bn)	4.03 (32.3%)	6.42 (33.5%)	5.92 (32.9%)	4.23 (33.5%)	8.88 (32.5%)	8.85 (32.5%)
Total CO_2 Emissions (Mt)	76 (32.2%)	50 (32.5%)	66 (32.1%)	48 (33.1%)	42 (33.0%)	39 (33.2%)
Carbon Intensity (gCO_2/kWh)	315.4 (32.3%)	182.1 (32.6%)	221.8 (32.3%)	168.5 (33.1%)	121.3 (33.0%)	104.1 (33.2%)
RE Generation Share	42.0% (32.5%)	52.7% (32.1%)	52.0% (31.8%)	61.4% (31.3%)	55.9% (31.6%)	61.7% (31.4%)
VRE Generation Share	25.1% (34.2%)	36.8% (33.2%)	34.7% (32.9%)	38.5% (32.2%)	35.9% (32.7%)	43.1% (32.0%)
VRE Curtailment	0.2% (352.5%)	0.1% (345.4%)	0.2% (336.8%)	1.2% (315.5%)	0.8% (320.9%)	0.8% (356.2%)
Interconnector Congestion	20.2% (37.2%)	9.6% (312.4%)	11.2% (310.6%)	18.1% (37.0%)	11.3% (311.6%)	6.6% (313.5%)
Total International Electricity Flow	10 TWh (34.1%)	28 TWh (35.5%)	34 TWh (34.0%)	37 TWh (33.6%)	28 TWh (35.8%)	17TWh (36.8%)

3.4. Results Overview: France

Table S5: Results overview for France

	2015 System	EU Reference 2030	ENTSOE Vision 1 2030	Vision 2 2030	Vision 3 2030	Vision 4 2030
Electricity Price (€/MWh)	38 (32.3%)	74 (31.7%)	59 (31.4%)	53 (32.5%)	60 (34.2%)	64 (35.0%)
Wind-weighted Price (€/MWh)	36 (3.2%)	70 (1.5%)	56 (1.8%)	49 (3.5%)	51 (5.6%)	55 (6.6%)
Solar-weighted Price (€/MWh)	34 (1.7%)	71 (1.1%)	54 (1.5%)	44 (3.2%)	39 (5.9%)	38 (6.5%)
Gas-weighted Price (€/MWh)	77 (14.7%)	98 (12.4%)	97 (12.5%)	93 (6.6%)	86 (3.4%)	89 (3.6%)
Coal-weighed Price (€/MWh)	44 (2.9%)	75 (1.1%)	64 (1.3%)	64 (2.1%)	72 (19.6%)	89 (23.5%)
Nuclear-weighted Price (€/MWh)	37 (2.0%)	74 (1.5%)	60 (1.3%)	53 (2.5%)	62 (3.9%)	66 (4.7%)
Total Generation Cost (€ bn)	2.97 (30.9%)	4.18 (31.6%)	3.27 (31.4%)	2.88 (31.4%)	3.59 (35.3%)	4.18 (35.8%)
Total CO_2 Emissions (Mt)	16 (33.8%)	29 (32.1%)	16 (33.7%)	10 (35.8%)	11 (39.2%)	13 (39.1%)
Carbon Intensity (gCO_2/kWh)	29.9 (33.8%)	46.6 (32.4%)	29.7 (33.9%)	18.5 (35.8%)	21.5 (39.2%)	25.8 (39.3%)
RE Generation Share	19.9% (30.9%)	32.8% (31.2%)	27.4% (31.2%)	25.1% (30.9%)	45.2% (31.3%)	46.4% (31.6%)
VRE Generation Share	5.4% (33.5%)	18.1% (32.5%)	13.1% (32.8%)	8.3% (32.9%)	26.2% (32.5%)	28.2% (32.8%)
VRE Curtailment	0.0% (30%)	0.0% (30%)	0.1% (356.6%)	0.5% (342.0%)	1.2% (323.5%)	1.2% (327.4%)
Interconnector Congestion	47.1% (31.8%)	46.3% (32.7%)	47.4% (32.5%)	41.3% (31.9%)	23.1% (34.8%)	22.7% (34.8%)
Total International Electricity Flow	47 TWh (31.1%)	81 TWh (32.7%)	92 TWh (32.4%)	75 TWh (31.5%)	57 TWh (35%)	46 TWh (35.2%)

3.5. Results Overview: Italy

Table S6: Results overview for Italy

	2015 System	EU Reference 2030	ENTSOE Vision 1 2030	Vision 2 2030	Vision 3 2030	Vision 4 2030
Electricity Price (€/MWh)	58 (30.9%)	85 (31.1%)	90 (31.0%)	83 (31.6%)	72 (32.2%)	76 (33.1%)
Wind-weighted Price (€/MWh)	55 (1.0%)	85 (1.4%)	85 (1.3%)	77 (2.1%)	64 (3.8%)	68 (3.1%)
Solar-weighted Price (€/MWh)	56 (1.2%)	82 (0.8%)	76 (1.4%)	65 (2.0%)	39 (3.9%)	44 (5.3%)
Gas-weighted Price (€/MWh)	60 (1.1%)	90 (2.0%)	108 (1.5%)	112 (1.7%)	94 (2.0%)	93 (2.2%)
Coal-weighed Price (€/MWh)	56 (0.9%)	86 (1.1%)	88 (1.0%)	84 (1.3%)	100 (22.7%)	110 (18.1%)
Nuclear-weighted Price (€/MWh)	0 (0%)	0 (0%)	0 (0%)	0 (0%)	0 (0%)	0 (0%)
Total Generation Cost (€ bn)	6.93 (30.8%)	11.7 (31.4%)	9.93 (31.0%)	7.18 (31.6%)	7.78 (32.3%)	10.09 (32.1%)
Total CO_2 Emissions (Mt)	97 (30.5%)	80 (31.0%)	94 (30.8%)	77 (31.3%)	38 (32.9%)	49 (32.5%)
Carbon Intensity (gCO_2/kWh)	362.3 (30.5%)	272.2 (30.9%)	305.8 (30.6%)	263.6 (30.9%)	129.0 (32.3%)	147.6 (32.0%)
RE Generation Share	41.2% (31.0%)	46.6% (31.3%)	49.5% (30.9%)	59.6% (30.8%)	55.9% (31.6%)	69.2% (31.0%)
VRE Generation Share	15.3% (32.7%)	21.5% (32.9%)	34.7% (32.9%)	21.3% (32.1%)	35.9% (32.7%)	30.3% (32.1%)
VRE Curtailment	0% (30%)	0% (30%)	0% (30%)	0.5% (320.8%)	4.2% (38.6%)	4.2% (37.8%)
Interconnector Congestion	48.9% (32.1%)	60.1% (32.4%)	48.1% (32.3%)	36.6% (34.3%)	37.8% (33.9%)	42.2% (4.2%)
Total International Electricity Flow	48 TWh (31.3%)	61 TWh (32.9%)	49 TWh (33.1%)	45 TWh (34.1%)	11 TWh (38.0%)	31TWh (36.3%)

3.6. Results Overview: Great Britain

Table S7: Results overview for Great Britain

	2015 System	EU Reference 2030	ENTSOE Vision 1 2030	Vision 2 2030	Vision 3 2030	Vision 4 2030
Electricity Price (€/MWh)	59 (31.6%)	79 (31.3%)	81 (31.2%)	55 (35.4%)	60 (34.5%)	61 (35.2%)
Wind-weighted Price (€/MWh)	56 (31.9%)	76 (31.3%)	77 (31.3%)	43 (36.7%)	47 (35.7%)	47 (36.3%)
Solar-weighted Price (€/MWh)	54 (32.9%)	78 (31.4%)	77 (31.9%)	54 (35.0%)	43 (35.8%)	47 (35.7%)
Gas-weighted Price (€/MWh)	67 (31.5%)	84 (31.8%)	86 (1.4%)	83 (4.1%)	87 (2.5%)	89 (2.7%)
Coal-weighed Price (€/MWh)	59 (31.6%)	80 (31.0%)	83 (31.3%)	78 (33.5%)	47 (35.7%)	0 (30%)
Nuclear-weighted Price (€/MWh)	58 (31.4%)	79 (31.2%)	80 (31.2%)	60 (33.7%)	66 (33.0%)	68 (33.5%)
Total Generation Cost (€ bn)	6.56 (31.5%)	8.82 (35.5%)	10.46 (32.8%)	3.56 (310.5%)	6.19 (37.9%)	6.60 (38.7%)
Total CO_2 Emissions (Mt)	146 (31.3%)	40 (36.0%)	70 (32.9%)	25 (310.6%)	28 (39.2%)	29 (39.8%)
Carbon Intensity (gCO_2/kWh)	523.2 (31.3%)	109.7 (36.0%)	249.9 (32.8%)	80.4 (311.8%)	76.6 (39.5%)	77.5 (310.2%)
RE Generation Share	22.2% (34.2%)	49.9% (33.4%)	32.9% (35.0%)	76.3% (32.9%)	65.6% (33.6%)	67.4% (33.6%)
VRE Generation Share	17.7% (35.6%)	31.9% (35.4%)	27.3% (36.0%)	64.3% (33.7%)	52.5% (34.7%)	56.6% (34.4%)
VRE Curtailment	0% (30%)	0% (30%)	0% (30%)	4.3% (315.7%)	2.7% (316.9%)	2.7% (318.0%)
Interconnector Congestion	35.9% (32.0%)	29.8% (33.2%)	39.9% (32.3%)	51.0% (32.3%)	36.3% (32.0%)	44.0% (1.6%)
Total International Electricity Flow	18 TWh (31.6%)	42 TWh (33.1%)	58 TWh (31.7%)	60 TWh (31.7%)	45 TWh (31.7%)	52TWh (31.5%)

4. WIND AND SOLAR OUTPUT VARIABILITY

This section shows the variability of annual capacity factors for wind and solar profiles by country for all scenarios considered. For all diagrams, the text on each country describes the mean capacity factor followed by the percentage point standard deviation over the course of all 30 weather years. The colour scale indicates the mean capacity factor for either wind or solar PV in each country.

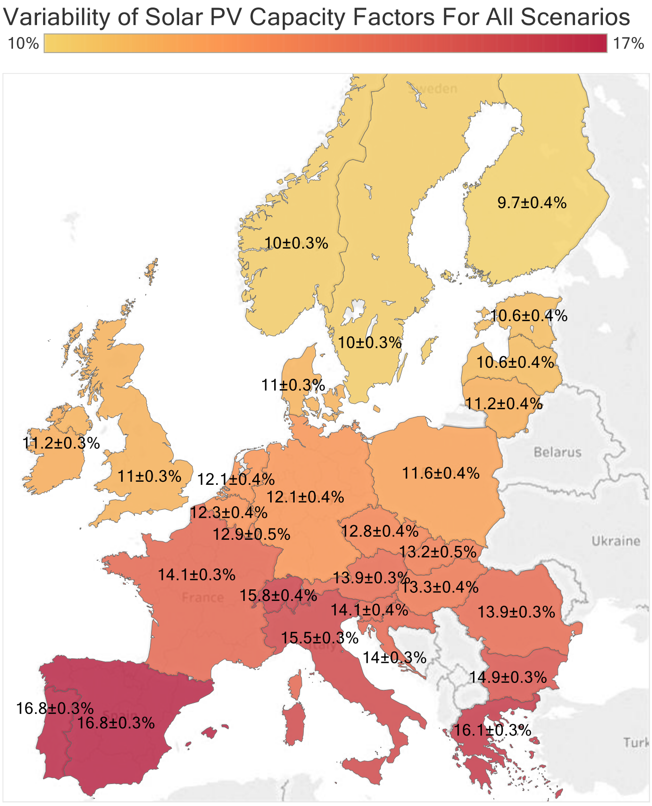

Figure S1: Solar capacity factor variability for all scenarios considered

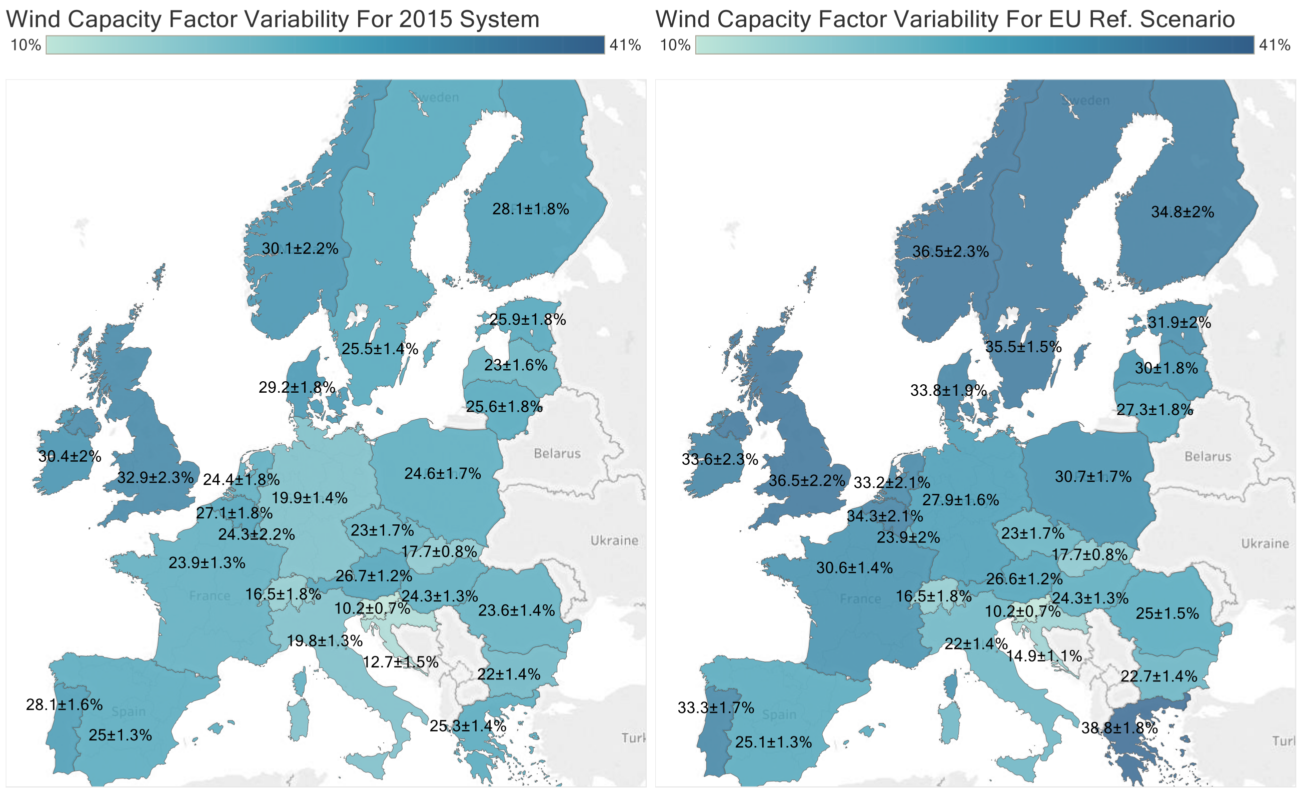

Figure S2: Wind capacity factor variability for 2015 System and EU Reference Scenario

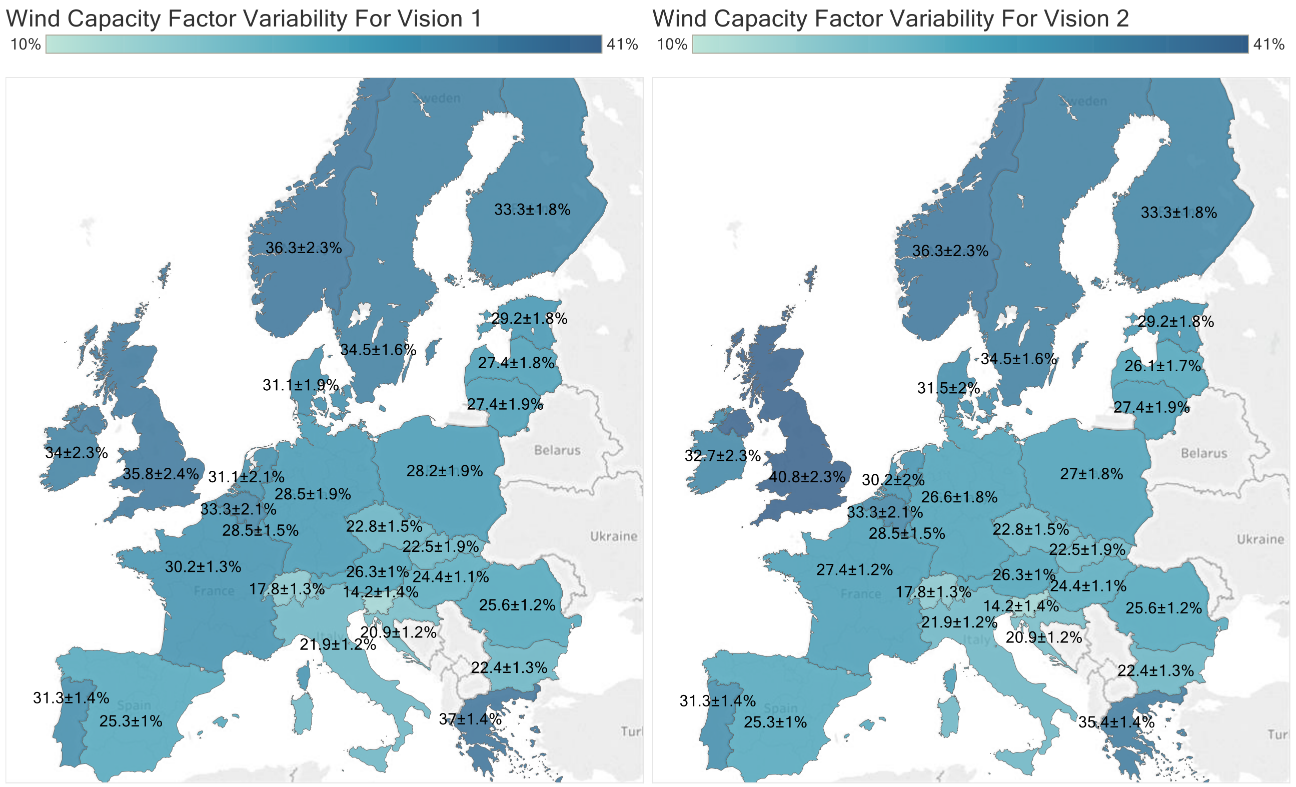

Figure S3: Wind capacity factor variability for Vision 1 and Vision 2

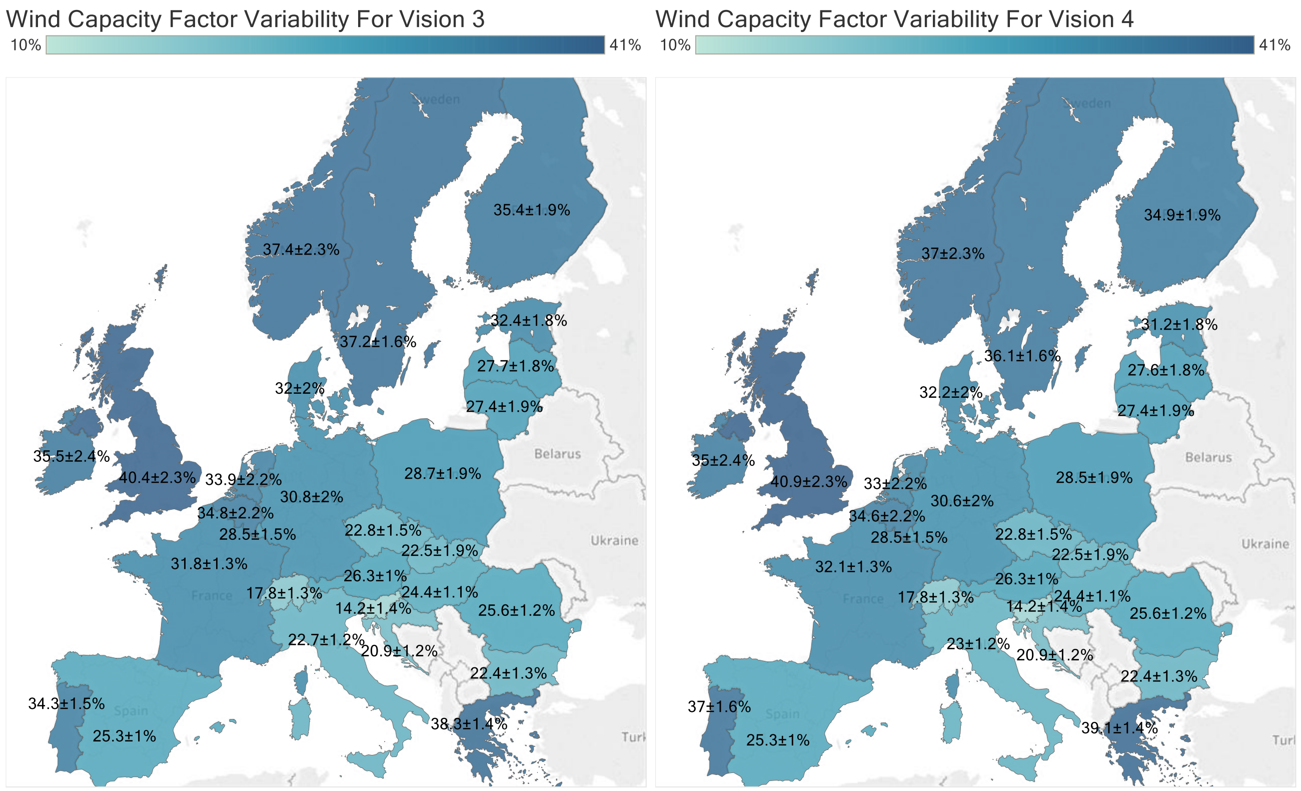

Figure S4: Wind capacity factor variability for Vision 3 and Vision 4

Supplemental information for Collins et al. (2018), doi: 10.1016/j.joule.2018.06.020

5. MOST REPRESENTATIVE SINGLE YEARS

The below table details the root mean squared error across eight metrics for all weather years considered. This error is determined as the RMS difference between each year and the long-run mean for each metric normalised by the long-run mean.

Table S8: Root mean squared error across various metrics for all weather years

Weather Year	VRE Penetration	CO_2 Emissions	Total Generation Costs	Market Prices	RE Penetration	IC Congestion	Total IC Flow	VRE Curtailment	Average RMS Error
1985	3.4%	3.7%	3.4%	1.2%	0.6%	2.2%	1.6%	3.3%	2.43%
1986	2.8%	2.6%	2.9%	1.2%	1.5%	0.7%	0.5%	12.3%	3.05%
1987	4.8%	4.8%	4.4%	1.9%	1.6%	1.7%	1.8%	12.3%	4.16%
1988	2.5%	2.7%	2.6%	1.2%	1.9%	1.2%	0.9%	3.9%	2.11%
1989	0.7%	0.6%	0.5%	0.9%	0.8%	1.0%	0.5%	0.9%	0.72%
1990	4.8%	4.7%	4.4%	2.8%	1.0%	2.4%	2.3%	24.2%	5.82%
1991	1.2%	1.3%	1.2%	1.0%	1.6%	0.3%	0.4%	9.2%	2.04%
1992	2.0%	1.8%	1.6%	1.1%	0.7%	0.7%	0.3%	16.4%	3.08%
1993	2.1%	2.2%	2.1%	1.5%	0.9%	1.2%	1.3%	7.3%	2.32%
1994	3.6%	3.4%	3.2%	2.8%	1.1%	1.5%	1.3%	19.2%	4.50%
1995	3.4%	3.3%	3.3%	2.5%	1.6%	1.9%	1.4%	1.3%	2.32%
1996	1.1%	1.4%	0.7%	1.0%	1.2%	0.7%	0.6%	14.4%	2.65%
1997	2.5%	2.8%	2.8%	1.3%	0.8%	0.9%	0.5%	8.5%	2.52%
1998	4.8%	4.9%	4.5%	2.9%	1.5%	1.8%	1.8%	12.3%	4.32%
1999	2.1%	1.8%	2.0%	1.4%	1.7%	1.1%	0.6%	3.7%	1.80%
2000	3.0%	2.9%	2.7%	1.4%	1.1%	2.2%	1.7%	2.0%	2.13%
2001	1.2%	1.3%	0.9%	0.5%	1.1%	1.1%	1.0%	4.9%	1.50%
2002	0.5%	0.7%	0.6%	0.6%	0.4%	0.7%	0.7%	4.3%	1.07%
2003	3.7%	3.9%	3.5%	2.1%	0.8%	2.4%	2.4%	9.6%	3.55%
2004	0.9%	0.9%	1.1%	0.7%	1.3%	0.6%	0.3%	7.4%	1.64%
2005	0.6%	0.8%	0.6%	0.5%	0.3%	0.5%	1.0%	6.3%	1.34%
2006	2.9%	2.7%	2.6%	2.0%	0.6%	1.6%	1.3%	8.4%	2.76%
2007	1.6%	1.7%	1.4%	1.4%	1.1%	2.0%	1.9%	2.8%	1.73%
2008	1.6%	2.1%	2.1%	1.7%	0.8%	0.7%	0.8%	4.3%	1.77%
2009	2.5%	2.6%	2.2%	1.3%	0.9%	1.8%	1.5%	9.3%	2.75%
2010	5.9%	5.6%	5.3%	3.1%	1.7%	2.2%	1.8%	26.1%	6.45%
2011	0.4%	0.3%	0.3%	0.6%	1.8%	0.5%	0.4%	7.3%	1.44%
2012	0.9%	0.6%	0.7%	0.6%	0.3%	0.4%	0.3%	3.7%	0.92%
2013	1.1%	1.3%	1.0%	0.5%	0.5%	1.1%	0.6%	3.9%	1.24%
2014	2.0%	1.5%	3.2%	8.1%	0.6%	0.5%	0.7%	12.0%	3.58%

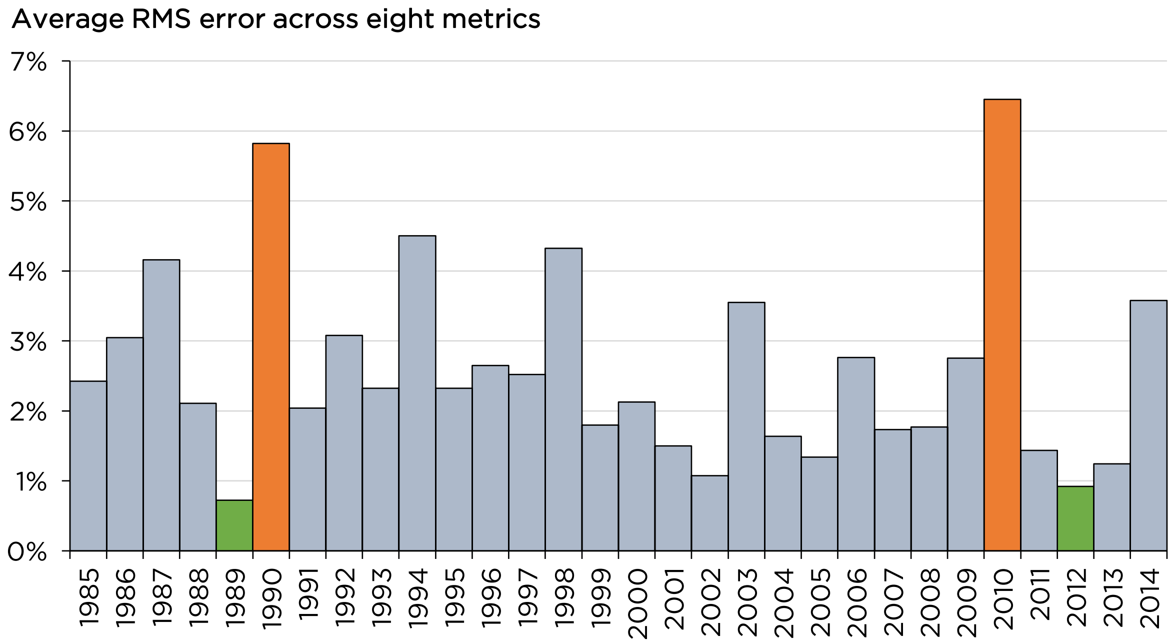

Figure S5: Average RMS Error across eight metrics

6. REFERENCES

1 Energy-Exemplar (2016). PLEXOS Integrated Energy Model. (http://energyexemplar.com/software/plexos-desktop-edition/).

2 Price Coupling of Regions (2016). EUPHEMIA: Description and Functioning, in: PCR (Ed.), EUPHEMIA Stakeholder Forum. EPEX Spot, Brussels. (http://static.epexspot.com/document/37602/Euphemia%20Public%20Presentation).

3 Pfenninger, S., and Staffell, I. (2016). Long-term patterns of European PV output using 30 years of validated hourly reanalysis and satellite data. Energy *114*, 1251-1265.

4 Staffell, I., and Pfenninger, S. (2016). Using bias-corrected reanalysis to simulate current and future wind power output. Energy *114*, 1224-1239.

5 Gelaro, R., McCarty, W., Suárez, M.J., Todling, R., Molod, A., Takacs, L., Randles, C.A., Darmenov, A., Bosilovich, M.G., Reichle, R., *et al.* (2017). The Modern-Era Retrospective Analysis for Research and Applications, Version 2 (MERRA-2). Journal of Climate *30*, 5419-5454.

6 Liléo, S., and Petrik, O. (2000). Investigation on the use of NCEP/NCAR, MERRA and NCEP/CFSR reanalysis data in wind resource analysis. sigma *1*.

7 Stickler, A., and Brönnimann, S. (2011). Significant bias of the NCEP/NCAR and twentieth-century reanalyses relative to pilot balloon observations over the West African Monsoon region (1940–1957). Quarterly Journal of the Royal Meteorological Society *137*, 1400-1416.

8 Staffell, I., and Green, R. (2014). How does wind farm performance decline with age? Renewable energy *66*, 775-786.
